# NanoASV: a snakemake workflow for reproducible field-based Nanopore full-length 16S metabarcoding amplicon data analysis

**DOI:** 10.1093/bioinformatics/btaf089

**Published:** 2025-03-20

**Authors:** Arthur Cousson, Frédéric Mahé, Ulysse Guyet, Damase Razafimahafaly, Laetitia Bernard

**Affiliations:** Eco&Sols, University of Montpellier, IRD, INRAe, CIRAD, Inst Agro, Montpellier F-34060, France; CIRAD, UMR PHIM, F-34398 Montpellier, France; PHIM, Univ Montpellier, CIRAD, INRAE, Institut Agro, IRD, Montpellier, France; Metabolic Genomics, Genoscope, Institut de Biologie François Jacob, CEA, CNRS, Université d'Evry, Université Paris Saclay, Evry 91000, France; Laboratoire des Radio-Isotopes, BP 3383, Route d’Andraisoro, Antananarivo 101, Madagascar; Eco&Sols, University of Montpellier, IRD, INRAe, CIRAD, Inst Agro, Montpellier F-34060, France

## Abstract

**Summary:**

NanoASV is a conda environment and snakemake-based workflow using state-of-the-art bioinformatics software to process full-length SSU rRNA (16S/18S) amplicons acquired with Oxford Nanopore Sequencing technology. Its strength lies in reproducibility, portability, and the possibility to run offline, allowing in-field analysis. It can be installed on the Nanopore MK1C sequencing device and process data locally.

**Availability and implementation:**

Source code and documentation are freely available at https://github.com/ImagoXV/NanoASV and Zenodo archive at https://doi.org/10.5281/zenodo.14730742.

## 1 Introduction

Oxford Nanopore Technologies (ONTs) offer an inexpensive and mobile solution for acquiring third-generation high-throughput sequencing data. However, their commercial computational solution, Epi2Me, suffers from several technical limitations (e.g. no taxonomy curation, unknown sequences grouping, and non-universal data format). Additionally, its dependency on internet connectivity and the transmission of large data volumes, particularly during the taxonomic affiliation step, make it challenging to use in remote areas with unreliable internet access. Only a few solutions addressing these challenges were published (Santos *et al.* 2020, Rodríguez-Pérez *et al.* 2021, Zorz *et al.* 2023).

Among these, NanoCLUST (Rodríguez-Pérez *et al.* 2021) is a reliable and efficient operational taxonomical unit-based analysis pipeline that offers species-level resolution. NanoCLUST does not seem to be maintained anymore, and the taxonomic recovery by SituSeq (Zorz *et al.* 2023) is hindered by a high prevalence of unknown classifications (see Supplementary Information S1). To overcome these limitations, we present NanoASV, a reference-based ASV workflow specifically designed for locally processing full-length SSU rRNA (16S/18S) metabarcoding sequencing data.

NanoASV is inspired by Nygaard *et al.*’s ASV-based pipeline (Nygaard *et al.* 2020) but aims to be more portable through encapsulation and more precise when processing unknown sequences.

## 2 Description

NanoASV uses a conda environment to ensure portability and reproducibility. By default, NanoASV accepts demultiplexed fastq files organized in barcode0 to barcodeXX sub-folders (e.g. “fastq_pass” if basecalled by the sequencing device), a CSV metadata file that is used to build a phyloseq object (*.Rdata* output file), and a CSV assignment file. NanoASV can also directly process unorganized fastq files (if they are found in the specified - -*dir* directory). In that context, if no metadata file is provided, NanoASV will generate a dummy one.

**Figure 1.**
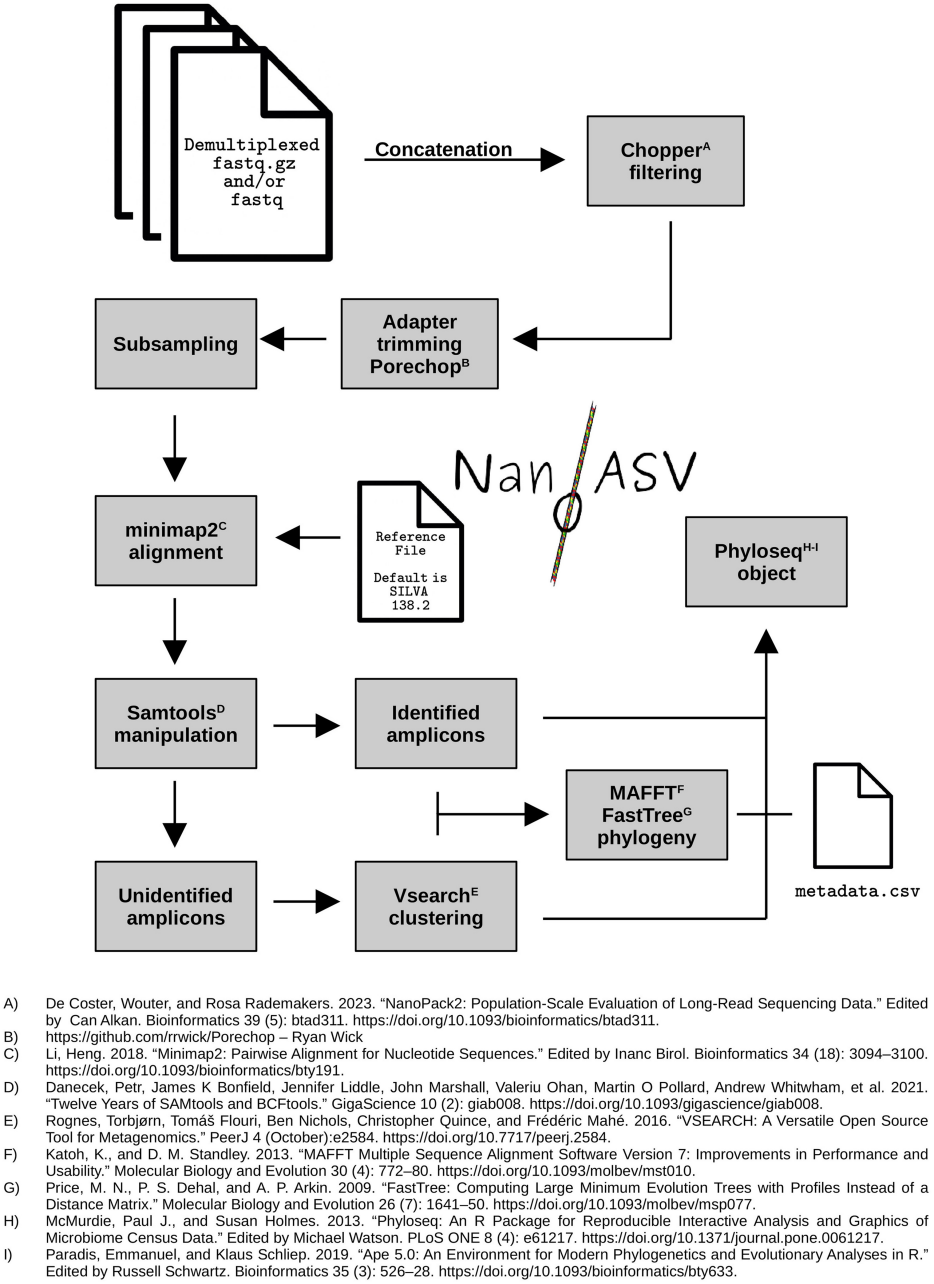
NanoASV workflow chart.

The workflow (summarized in Fig. 1) processes both compressed and uncompressed fastq files, which are typically 4000 sequences long. It groups sequences by sample, using barcode identifiers. Subsequently, the sequences are filtered using Chopper (De Coster and Rademakers 2023) with specific parameters (*- -quality 8, - -minlength 1400 - -maxlength 1700* by default) to remove low-quality reads. Porechop (https://github.com/rrwick/Porechop—Ryan Wick) is employed to identify and remove sequencing adapters from the filtered sequences.

By default, fastq files are subsampled to 50 000 sequences per barcode, but this can be changed with the option - -*subsampling*. These subsampled sequences are then aligned with minimap2 (Li 2018) (*- -model map-ont* by default) against a user-provided reference fasta file (see README file for reference requirements), or by default against SILVA138.2 (Quast *et al.* 2013). Unmapped reads, secondary alignments, and supplementary alignments are removed using samtools (*samtools view -F 4 -F 256 -F 272 -F 2048 -F 2024*) (Danecek *et al.* 2021) to only keep correctly mapped reads. Assignment data and statistics are written to flat .csv files. Mapping quality threshold (MapQ) can be adjusted with - -*samtools-qual* (default 0, see Supplementary Information S3).

One of the key innovations of NanoASV lies in its treatment of unknown sequences (see Supplementary Information S2). As an example, with Epi2Me or SituSeq (Zorz *et al.* 2023), unassigned sequences are typically assigned the label “Unknown” without extracting any additional information. This approach is problematic because it does not differentiate between singletons or rare erroneous sequences and abundant potentially meaningful DNA sequences within the dataset. To address this challenge, we have implemented a vsearch-based clustering step (Rognes *et al.* 2016) specifically for unassigned sequences. To account for the high error rate associated with ONT data, vsearch is set to use a low clustering similarity threshold (*- -id 0.7* by default). Unknown clusters with a total abundance lower than 5 are excluded from the final results.

Reference and unknown consensus sequences are pooled and aligned with MAFFT (Katoh and Standley 2013). A 16S-based phylogenetic tree is computed with FastTree (Price *et al.* 2009).

Abundance tables of assigned and unassigned clusters, taxonomy table, and 16S-based phylogeny are used to produce a phyloseq object. NanoASV expects to find a metadata.csv file in the same directory as the demultiplexed barcodes (location controlled by the option - -*metadata*). Using *ape* and *phyloseq* R packages (McMurdie and Holmes 2013, Paradis and Schliep 2019), NanoASV outputs a readily usable phyloseq object containing comprehensive information for both assigned and unknown clusters, as well as an abundance and taxonomy table (csv), and a phylogenetic tree. Sequences assigned to Eukaryota, Chloroplast, and Mitochondria are removed by default from the phyloseq object. This can be modified with the - -*no-r-cleaning* option.

Benchmarking NanoASV against the SituSeq and Nygaard *et al.* pipelines showed that it is faster and more memory-efficient while recovering similar alpha diversity trends and taxonomic profiles (see Supplementary Information S1).

## 3 Installation and usage

NanoASV is easy to install. Detailed instructions can be found in the README file: (i) clone the github repository; (ii) run the installation script; and (iii) activate the conda environment and run

*nanoasv - -dir path/to/dir - -out path/to/output [- -options]*

A self-test with a small dataset can be run with *nanoasv - -mock*, producing a typical NanoASV analysis output. All available options are detailed on the GitHub README file.

NanoASV runs on GNU/Linux x86-64 and Aarch64 systems, as well as on the Nanopore MK1C sequencing device (Aarch64—Minion MK1C version).

## 4 Conclusion

NanoASV is an efficient and reliable local container-based solution to process Nanopore metabarcoding amplicon data. Its innovative approach to handling unassigned sequences allows users to discover potentially new molecular diversity. Its portability allows it to be installed directly on the Nanopore MK1C sequencing device and to process data locally, making it suited for offline usage and in-field analysis.

## Supplementary Material

btaf089_Supplementary_Data

## Data Availability

The benchmark data underlying this article are available in Zenodo, at https://doi.org/10.5281/zenodo.14979767
