# Supplementary material for: NanoASV: a snakemake workflow for reproducible field-based Nanopore full-length 16S metabarcoding amplicon data analysis
Source: Bioinformatics. 2025 Mar 20;41(3):btaf089. doi: 10.1093/bioinformatics/btaf089 (PMC11937976; doi:10.1093/bioinformatics/btaf089)
Supplement: btaf089_Supplementary_Data [file btaf089_supplementary_data.zip › SUPP_INFO_01_NanoASV_benchmarking.pdf]

Pipelines benchmarking

Table of Contents

Pipelines benchmarking....1
Nygaard dataset....2
Alpha Diversity....2
Taxonomical profile....3
Pipelines specs comparisons....4
Conclusion....4
Bibliography....5
Scripts....6
NanoASV....6
NanoASV.sh....6
SituSeq....6
SituSeq.sh....6
setup.r....6
preprocessing.r....7
Stream-1A.r....8
Stream-1B.r....9
Phylosequization.r....11
Nygaard Manual Pipeline....12
Nygaard.sh....12
Abundance_tables_generation.sh....12
formating.sh....13
Phylosequization.R....13
Pipeline comparisons....16
Pipeline_comparisons.r....16

Nygaard dataset

Nota Bene : We could not never run NanoCLUST, hence it's absence from the benchmarking. Scripts used to produce this analysis are present in the end of this document.

We ran (Nygaard et al. 2020) dataset with three different software solution : SituSeq (Zorz et al. 2023), Nygaard manual pipeline (Nygaard et al. 2020) and NanoASV. It should be noted that NanoASV is the only one that outputs a phyloseq (McMurdie and Holmes 2013) object. The two others require manual file manipulation to achieve the same results. NanoASV can natively output a 16S phylogeny thanks to MAFFT (Katoh and Standley 2013) and FastTree (Price, Dehal, and Arkin 2009). The same reference dataset was used in each pipeline : DADA2 maintained training set silva_nr99_v138.1_train_set.fa. We made this choice as it was the easiest reference to use with SituSeq. Random UUID were assigned to each reference sequence to make it work with NanoASV.

Alpha Diversity

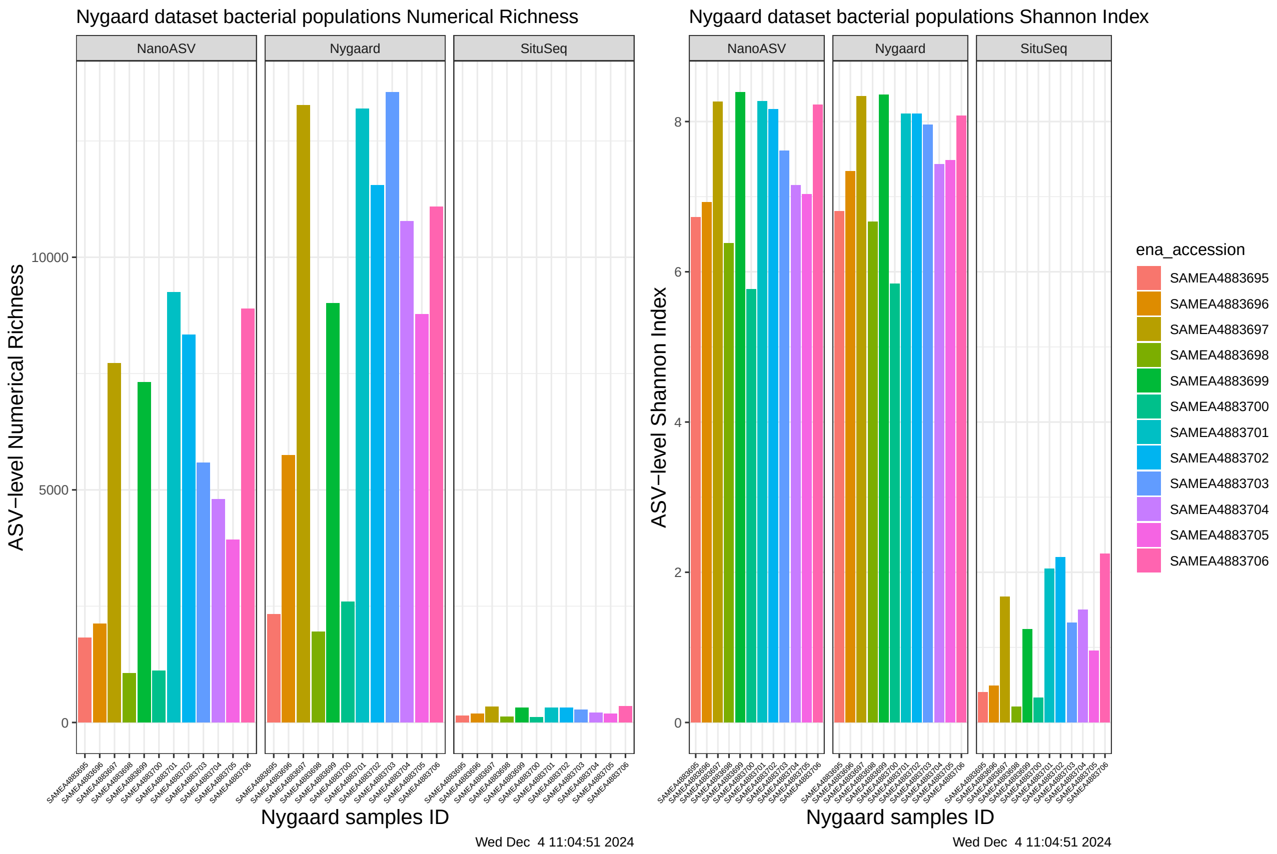

Figure 1: Benchmarking - Alpha diversity - Nygaard dataset

We see in Sup. Fig 1 that NanoASV and Nygaard pipeline outputs show similar trends in matter of numerical richness and Shannon index. Despite lower values, the same trends are observed with SituSeq. This is shown in Supp Table 1.

	NanoASV shannon	Nygaard shannon	SituSeq shannon
NanoASV shannon	1	0.9726359	0.8631368
Nygaard shannon	0.9726359	1	0.8057876
SituSeq shannon	0.8631368	0.8057876	1

	NanoASV richness	Nygaard richness	SituSeq richness
NanoASV richness	1	0.8729914	0.9734502
Nygaard richness	0.8729914	1	0.8679892
SituSeq richness	0.9734502	0.8679892	1

Table 1: Alpha Diversity measures correlation matrix between different pipelines

Taxonomical profile

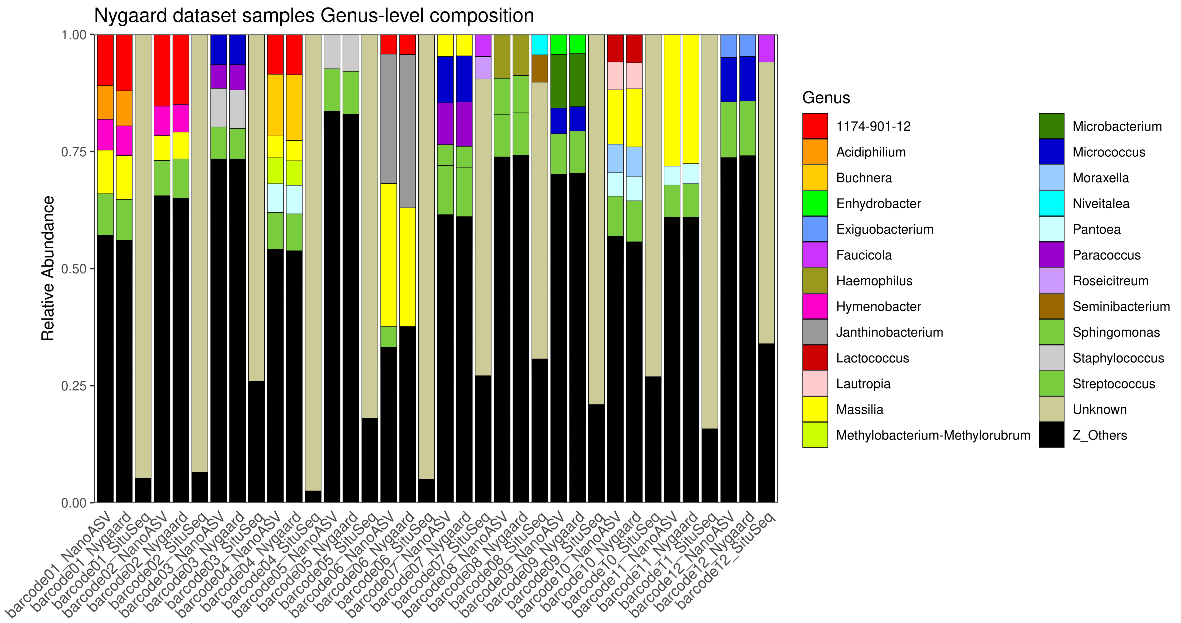

Figure 2: Genus-level composition stack plot. Nygaard et al dataset wit three different bioinformatics pipeline : NanoASV, Nygaard published pipeline and SituSeq

We see with Sup. Fig 2 that Genus level taxonomical profile looks very similar between NanoASV and Nygaard pipeline. SituSeq had difficulties recovering a precise taxonomical profile with unassigned sequences representing 59 to 97% of a sample total taxonomical assignments. While NanoASV and Nygaard pipelines both assigned 100% of sequences against the silva_nr99_v138.1_train_set.fa reference dataset. Note that full silva 138.2 reference does never allow 100% reads assignements. A Mantel test (vegan::mantel() with 999 permutations) was performed to compare different pipelines Bray-Curtis dissimilarity matrices. NanoASV and Nygaard pipeline showed high similarity (Mantel statistic r: 0.8735 – Significance: 0.001). NanoASV and SituSeq showed about half this value (Mantel statistic r: 0.5459 – Significance: 0.005). Nygaard and SituSeq showed the lowest similarity (Mantel statistic r: 0.3464 – Significance: 0.021)

Pipelines specs comparisons

Reference	Dataset	Machine	Allocated threads	Software solution	Elapsed (wall clock) Time (hh:mm)	Maximum resident Set size (kbytes)	Maximum resident Set size (Gbytes)
nr99_v138.1_train_set	Mock (2 barcodes)	PC – i5 32 Gb	2	**NanoASV**	**00:02**	**3397720**	**3.3977**
				**SituSeq**	**00:05**	**4675464**	**4.6755**
				**Nygaard**	**00:07**	**3541348**	**3.5413**
	Nygaard dataset	Slurm cluster 200Gb	24	**NanoASV**	**00:55**	**15813096**	**15.8131**
				**SituSeq**	**20:30**	**22795204**	**22.7952**
				**Nygaard**	**06:28**	**16254344**	**16.2543**

Table 2: Pipeline specs comparisons

Specs on personal computer were obtained with *usr/bin/time -v* : Max Resident Set Size. On slurm cluster, maximum resident size was obtained through slurm command *sacct.* Multi-threaded jobs can be hard to track for memory consumption. Peak memory values are probably underestimated.

Table 2 shows that NanoASV is faster and more memory efficient than Nygaard pipeline and SituSeq.

Conclusion

SituSeq showed to be very different in term of output when compared to Nygaard pipeline and NanoASV. SituSeq did not recover an extensive taxonomical profile, but global trends still similar.

NanoASV shows very similar trends as Nygaard pipeline, in matter of alpha diversity and taxonomical profile. NanoASV appeared around 6 times faster and more memory efficient than Nygaard manual pipeline.

Bibliography

Katoh, K., and D. M. Standley. 2013. "MAFFT Multiple Sequence Alignment Software Version 7: Improvements in Performance and Usability." *Molecular Biology and Evolution* 30 (4): 772–80. https://doi.org/10.1093/molbev/mst010.

McMurdie, Paul J., and Susan Holmes. 2013. "Phyloseq: An R Package for Reproducible Interactive Analysis and Graphics of Microbiome Census Data." Edited by Michael Watson. *PLoS ONE* 8 (4): e61217. https://doi.org/10.1371/journal.pone.0061217.

Nygaard, Anders B., Hege S. Tunsjø, Roger Meisal, and Colin Charnock. 2020. "A Preliminary Study on the Potential of Nanopore MinION and Illumina MiSeq 16S rRNA Gene Sequencing to Characterize Building-Dust Microbiomes." *Scientific Reports* 10 (1): 3209. https://doi.org/10.1038/s41598-020-59771-0.

Price, M. N., P. S. Dehal, and A. P. Arkin. 2009. "FastTree: Computing Large Minimum Evolution Trees with Profiles Instead of a Distance Matrix." *Molecular Biology and Evolution* 26 (7): 1641–50. https://doi.org/10.1093/molbev/msp077.

Zorz, Jackie, Carmen Li, Anirban Chakraborty, Daniel A Gittins, Taylor Surcon, Natasha Morrison, Robbie Bennett, Adam MacDonald, and Casey R J Hubert. 2023. "*SituSeq* : An Offline Protocol for Rapid and Remote Nanopore 16S rRNA Amplicon Sequence Analysis." *ISME Communications* 3 (1): 33. https://doi.org/10.1038/s43705-023-00239-3.

Scripts

NanoASV

NanoASV.sh

```
#!/bin/bash
#SBATCH -p unlimitq
#SBATCH -t 12:00:00
#SBATCH --mem=200G
#SBATCH --cpus-per-task=24

source ~/work/miniconda3/bin/activate

conda activate NanoASV

DATABASE="/home/acousson/work/NanoASV/Benchmark/NR99_ref/NanoASV_format_silva_nr99_v138.1_train_set.fa"

/usr/bin/time -v bash ${NANOASV_PATH}/workflow/run.sh --dir ~/work/Data/Nygaard/ --out nanoasv.out --num-
process 24 --database ${DATABASE} --notree
```

SituSeq

SituSeq.sh

```
#!/bin/bash
#SBATCH -p unlimitq
#SBATCH -t 24:00:00
#SBATCH --mem=200G
#SBATCH --cpus-per-task=24

module load statistics/R/4.4.0

/usr/bin/time -v Rscript ~/work/NanoASV/Benchmark/SituSeq/scripts/setup.r
/usr/bin/time -v Rscript ~/work/NanoASV/Benchmark/SituSeq/scripts/preprocessing.r
/usr/bin/time -v Rscript ~/work/NanoASV/Benchmark/SituSeq/scripts/Stream-1A.r
/usr/bin/time -v Rscript ~/work/NanoASV/Benchmark/SituSeq/scripts/Stream-1B.r
/usr/bin/time -v Rscript ~/work/NanoASV/Benchmark/SituSeq/scripts/Phylosequization.r
```

setup.r

```
#install tidyverse
install.packages("tidyverse")
#install ShortRead
if (!require("BiocManager", quietly = TRUE))
  install.packages("BiocManager")
BiocManager::install("ShortRead")
#install dada2 (https://benjjneb.github.io/dada2/dada-installation.html)
if (!require("BiocManager", quietly = TRUE))
  install.packages("BiocManager")
BiocManager::install("dada2")
install.packages("installr")
library(installr)
```

preprocessing.r

```
##This code can be copied and pasted directly into R to concatenate, filter, and trim Nanopore 16S rRNA
gene sequences.
##The working directory folder should be set to the folder containing the subfolders of fastq files from
each barcoded sample. E.g From the Nanopore default output, the "fastq_pass" folder would be the working
directory
##Each subdirectory should start with "barcode". If you would like to add extra identifying text, please
add it to the subdirectory with an underscore after "barcode". E.g. "barcode01_sample1"
##The end result of this code will be filter and trimmed sequences and a csv file containing information
on the reads in and out
##You can change the parameters in the following section before running the code


######
#parameters to set before running:
path_to_working_directory = "/home/acousson/work/NanoASV/Benchmark/SituSeq/Dataset/" #leave as a "." if
you want to set your working directory manually in RStudio "Session"--> "Set Directory" --> "Choose
Directory"
#parameters for the filterAndTrim command:
minLength = 1200 #Removes reads shorter than this length. Minimum length is enforced AFTER trimming
maxLength = 1800 #Removes reads longer than this length. Maximum length is enforced BEFORE trimming
trimLeft = 100 #The number of nucleotides to remove from the start of each read. Must cover the primer
length
trimRight = 100 #The number of nucleotides to remove from the end of each read. Must cover the primer
length

######
#in R
#set working directory if you haven't set it manually
setwd(path_to_working_directory)

#load packages (in this order to avoid masking issues)
library(ShortRead)
library(dada2)
library(tidyverse)

#Concatenate nanopore files in R
folders <- list.files(pattern = "barcode" )

for (directory in folders) {
  print(directory)
  files = list.files(path = paste(directory, "/", sep = ""), pattern = ".fastq*")
  print(files)
  fout = file.path(paste(directory, "combined.fastq.gz", sep = "_"))
  for (fl in files) {
    fq = readFastq(paste(directory,"/",fl, sep = ""))
    writeFastq(fq, fout, mode="a")
  }
}


#save path to object
path = getwd()

#Forward and fastq filenames have format:
#barcode01_combined.fastq
fnFs = sort(list.files(path, pattern=".fastq.gz", full.names = TRUE))

#Extract sample names, assuming filenames have format: #samplename_XXX.fastq
```

```
sample.names = sapply(strsplit(basename(fnFs), "\\."), `[`, 1)

#filter and trim reads- create new paths for new files
filtFs <- file.path(path, "filtered", paste0(sample.names, "_filt.fastq"))
names(filtFs) = sample.names

#filter and trim command - will create new fastq files with filtered and trimmed sequences
out = filterAndTrim(fnFs, filtFs, trimLeft = trimLeft, trimRight = trimRight, maxLen = maxLength, minLen =
minLength,  truncQ = 0, compress = FALSE)

#see how many reads were lost and write to csv file
head(out,12)
write.csv(out, "Filtered_sequence_summary.csv")
```

Stream-1A.r

```
##This code is meant to be run after completing the Preproccessing.R code on your raw fastq files. After
the Preprocessing.R code you will have filtered and trimmed fastq files for each sample in your analysis.
##This code can be copied and pasted directly into R to assign taxonomy to full length 16S rRNA reads
sequenced using the Nanopore MinION platform and 16S barcode kit.
##The working directory folder should be set to the folder containing subfolders of fastq files from each
barcoded sample. E.g From the Nanopore default output, the "fastq_pass" folder would be the working
directory (same as for the Preprocessing step)
##The end result of this code will be a series of csv files containing the taxonomic information for each
Nanopore sequence
#Use Stream 1B to visualize this data (bubble plot with abundance of all taxa, and a bar plot with the
abundance of the top 10 taxa per sample)
##You can change the parameters in the following section before running the code

######
#parameters to set before running
subsample_depth = 50000 #each sample will be randomly subsampled to this number of reads, prior to
taxonomic assignment (after filtering and trimming). For no subsampling see Nanopore_no_rarefaction.R
under "backups"
path_to_taxonomy_database =
"/home/acousson/work/NanoASV/Benchmark/NR99_ref/silva_nr99_v138.1_train_set.fa" #change to location of
taxonomy database in relation to working directory (easiest to copy taxonomy database to working
directory)
path_to_working_directory = "/home/acousson/work/NanoASV/Benchmark/SituSeq/Dataset/" #leave as a "." if
you want to set your working directory manually in RStudio "Session"--> "Set Directory" --> "Choose
Directory"
minBoot = 50 #Set the minBoot parameter for assignTaxonomy. minBoot refers to the minimum bootstrapping
support required to return a taxonomic classification. Choose a number between 0-100, with 100 being the
most stringent.
######

#in R
#set working directory
setwd(path_to_working_directory)

#load packages in this order to avoid masking issues
library(ShortRead)
library(dada2)
library(tidyverse)

#save path to object
path<-path_to_working_directory
```

```
#fastq filenames have format:
#barcode01_combined.fastq
fnFs = sort(list.files(path, pattern=".fastq.gz", full.names = TRUE))

#extract sample names, assuming filenames have format: #samplename_XXX.fastq
sample.names = sapply(strsplit(basename(fnFs), "\\."), `[`, 1)

#path for filtered and trimmed reads
filtFs <- file.path(path, "filtered", paste0(sample.names, "_filt.fastq"))
names(filtFs) = sample.names

#import sequences and assign taxonomy - with subsetting to subsampling depth
#this will create a csv file for each sample with the sequence and its assigned taxonomy
for (fastq in filtFs) {
  print(fastq)
  seqs = getSequences(fastq)
  sub = sample(1:length(seqs), subsample_depth, replace=FALSE)
  seq2 = seqs[sub]
  tax_rc = assignTaxonomy(seq2, path_to_taxonomy_database, multithread=TRUE, tryRC = TRUE, minBoot =
minBoot)
  base = basename(fastq)
  samples = gsub("_filt.fastq", "", base)
  write.csv(tax_rc, paste('tax', samples, 'csv', sep = '.' ))
```

Stream-1B.r

```
  ##This code is meant to be run after completing the Preproccessing.R and Stream1A_assignTaxonomy.R code
on your raw fastq files. After the Stream1A_assignTaxonomy.R code you will have a csv file for each sample
containing taxonomic information for the Nanopore sequences.
  ##This code can be copied and pasted directly into R to summarize visualize the taxonomy assigned in
Stream1A_assignTaxonomy.R
  ##The working directory folder should be set to the folder containing subfolders of fastq files from
each barcoded sample. E.g From the Nanopore default output, the "fastq_pass" folder would be the working
directory (same as for the Preprocessing and Stream1A steps)
  ##The end result of this code will be a bubble plot with abundance of all taxa, and a bar plot with the
abundance of the top 10 taxa per sample
  ##You can change the parameters in the following section before running the code



  ######
#parameters to set before running
taxonomic_level = "Genus" #choose from "Phylum" "Class" "Order" "Family" "Genus"
sample_number = 12
path_to_working_directory = "/home/acousson/work/NanoASV/Benchmark/SituSeq/Dataset/" #leave as a "." if
you want to set your working directory manually in RStudio "Session"--> "Set Directory" --> "Choose
Directory"
######

#in R
#set working directory
setwd(path_to_working_directory)

#load tidyverse package
library(tidyverse)


#read in newly made csv files
temp = list.files(pattern="tax.*.csv")
temp_list = list()
```

```
for (i in 1:length(temp)) {
  sample = gsub(".csv", "", temp[[i]])
  sample2 = gsub("tax.","",sample)
  new = read.csv(temp[i], header = TRUE)
  new2 = new %>% filter(Kingdom == "Bacteria") %>% select(all_of(taxonomic_level)) %>% group_by_all() %>%
summarise(n = n()) %>% mutate(abund = n/(colSums(as.matrix(n)))*100) %>% select(-n)
  colnames(new2) = c(taxonomic_level, sample2)
  temp_list[[length(temp_list) + 1]] <- new2 }


#merge all data frames in list
tax_df = temp_list %>% reduce(full_join, by=taxonomic_level)

#remove "_combined" from sample name
colnames(tax_df) = gsub("_combined","",colnames(tax_df))

#write summary csv of taxonomic level
write.csv(tax_df, paste0(taxonomic_level,"_summary.csv"), row.names = FALSE)

#convert data to long format
tax_df_long = tax_df %>% pivot_longer(!taxonomic_level, names_to = "Sample", values_to = "Abundance")

#colour scheme for bubble plot
colours = colorRampPalette(c("#2F4858", "#33658A", "#86BBD8", "#830689", "#F5A614", "#F26419", "#BB3551",
"#C1D7AE", "#68AC5D", "#EBDDAD"))(sample_number)


#bubble plot
xx = ggplot(tax_df_long, aes(x = Sample, y = reorder(get(taxonomic_level), desc(get(taxonomic_level))))) +
geom_point(aes(colour = Sample, size= Abundance), alpha = 0.7) +theme(legend.key = element_blank(),
legend.title = element_text(size = 10), panel.border = element_rect(fill = NA, colour = "grey80"),
axis.text.y = element_text(size = 7), axis.text.x = element_text(size = 7, angle = 90, vjust = 0.3, hjust
=1), panel.background = element_blank(), panel.grid.major = element_line(colour = "grey94")) +
scale_radius(range=c(1,8), breaks = c(1,10,30,50)) + labs(x = "", y = "", colour = taxonomic_level) +
scale_colour_manual(values = colours) + guides(colour = "none")
xx

#save bubble plot
ggsave(paste0("bubble_plot_",taxonomic_level,".png"), height = 9, width = 5.5)

#select top 10 most abundant taxa, based on maximum abundance in data set
tax_df$max = apply(tax_df[,2:ncol(tax_df)], 1, FUN = max, na.rm = TRUE)
tax_df2 <- tax_df[order(-tax_df$max),][1:10,]

#colour scheme for bar plot
colours = c("#2F4858", "#33658A", "#86BBD8", "#830689", "#F5A614", "#F26419", "#BB3551",  "#C1D7AE",
"#68AC5D", "#EBDDAD")

#convert data to long format
tax_df2_long = tax_df2 %>% select(-max) %>% pivot_longer(!taxonomic_level, names_to = "Sample", values_to
= "Abundance")

#remove "_combined" from sample name
tax_df2_long$Sample = gsub("_combined","",tax_df2_long$Sample)

#bar plot of most abundant taxa
gg = ggplot(tax_df2_long, aes(x = Sample, y = Abundance)) + geom_bar(aes(fill = get(taxonomic_level)),
colour = "black", position = "stack", stat = "identity") + scale_fill_manual(values = colours) + labs(x =
"", y = "Relative Abundance (%)", fill = taxonomic_level) + theme(panel.background = element_blank(),
```

```
panel.border = element_rect(fill =NA, colour = "black"), axis.text.x = element_text(angle = 90, hjust = 1,
vjust = 0.3), legend.key = element_blank()) + scale_y_continuous(limits = c(0,100), expand = c(0,0))
gg

#save plot
ggsave(paste0("bar_plot_top_",taxonomic_level,".png"), height = 6, width = 5)
```

Phylosequization.r

```
#SituSeq Phylosequization

library(phyloseq)

setwd("/home/acousson/work/NanoASV/Benchmark/SituSeq/Dataset/")

metadata <- read.csv("metadata.csv", row.names = 1 )

OTU <- read.csv("Genus_summary.csv")

TAX1 <- read.csv("tax.barcode01.csv")
TAX2 <- read.csv("tax.barcode02.csv")
TAX3 <- read.csv("tax.barcode03.csv")
TAX4 <- read.csv("tax.barcode04.csv")
TAX5 <- read.csv("tax.barcode05.csv")
TAX6 <- read.csv("tax.barcode06.csv")
TAX7 <- read.csv("tax.barcode07.csv")
TAX8 <- read.csv("tax.barcode08.csv")
TAX9 <- read.csv("tax.barcode09.csv")
TAX10 <- read.csv("tax.barcode10.csv")
TAX11 <- read.csv("tax.barcode11.csv")
TAX12 <- read.csv("tax.barcode12.csv")

TAX <- rbind(TAX1, TAX2, TAX3, TAX4, TAX4, TAX5, TAX6, TAX7, TAX8, TAX9, TAX10, TAX11, TAX12)

TAX <- TAX[,-1]

TAX <- unique(TAX)

TAX <- rbind(TAX, rep("Unknown", 6))

OTU[is.na(OTU)] <- 0

OTU[OTU$Genus == "0",1] <- "Unknown"

TAXOTU <- merge(TAX, OTU, by = "Genus")

rownames(TAXOTU) <- uuid::UUIDgenerate(n=nrow(TAXOTU))

SituSeq.Nygaard <- phyloseq(otu_table(TAXOTU[,7:ncol(TAXOTU)], taxa_are_rows = T),
                        tax_table(as.matrix(TAXOTU[,1:6])),
                        sample_data(metadata))

save(SituSeq.Nygaard, file = "SituSeq_Nygaard.rdata")
```

Nygaard Manual Pipeline

Nygaard.sh

```
#!/bin/bash
#SBATCH -p unlimitq
#SBATCH -t 72:00:00
#SBATCH --mem=200G
#SBATCH --cpus-per-task=24

module load bioinfo/Porechop/0.2.4

DATABASE="/home/acousson/work/NanoASV/Benchmark/NR99_ref/NanoASV_format_silva_nr99_v138.1_train_set.fa"

DATASET="/home/acousson/work/NanoASV/Benchmark/Nygaard/Dataset"

#Filtering
for barcode in ${DATASET}/*.fastq.gz; do
   /usr/bin/time -v porechop --verbosity 0 -i ${barcode} -o "choped_$(basename "$barcode")"
       echo $barcode
done

#module purge

module load devel/Miniconda/Miniconda3
module load bioinfo/chopper/0.7.0
#Choping

for barcode in ./choped*.fastq.gz; do
    /usr/bin/time -v zcat $barcode | chopper -l 1300 --maxlength 1700 -q 9 > filtered_$(basename $
{barcode}).fastq
done

module purge
module load bioinfo/LAST/1542

#Index
/usr/bin/time -v lastdb Nr_test_99.db ${DATABASE}
#Alignment
for barcode in ./filtered*.fasta; do
   /usr/bin/time -v  lastal -v Nr_test_99.db ${barcode} -f BlastTab -P 8 -r 1 -q 1 -a 1 -b 1 > $
{barcode}.blast.tab
done

bash Abundance_tables_generation.sh
bash formating.sh
bash Phylosequization.R
```

Abundance_tables_generation.sh

```
#!/bin/bash

for file in ./*.blast.tab; do
cut -f2 $file | sort | uniq -c > ASV_TABLE_$(basename $file)
done
```

formating.sh

```
#!/bin/bash

for file in ./*ASV_*; do
sed -i 's/^[[:space:]]*//' $file
done
```

Phylosequization.R

```
#NanoASV phylosequisation
#Arthur Cousson - 2023
#Contact : arthur.cousson@ird.fr
# Nygaard pipeline output phyloseq generation

setwd("~/Software_dev/Benchmark_Nygaard/Nygaard_pipeline_analysis/")

#Phylosequization -----
library(phyloseq)
library(ape) #To handle trees
metadata <- read.csv("metadata.csv", row.names = 1, header = TRUE, check.names = FALSE)
#metadata <- read.csv(paste0(METADATA,"/metadata.csv"), row.names = 1, header = TRUE, check.names = FALSE)
barcodes <- rownames(metadata)

##ASV tables ----
#Individuals ASV tables loading
temp_ASV = list.files(path = "./", pattern = "*.tsv")


temp_ASV <- list()
temp_ASV[[1]] <- read.csv("ASV_TABLE_filtered_choped_barcode01.fasta.blast.tab.tsv", check.names = F, sep
= "\t", header = F, row.names = 1)
temp_ASV[[2]] <- read.csv("ASV_TABLE_filtered_choped_barcode02.fastq.gz.fasta.blast.tab.tsv", check.names
= F, sep = "\t", header = F, row.names = 1)
temp_ASV[[3]] <- read.csv("ASV_TABLE_filtered_choped_barcode03.fastq.gz.fasta.blast.tab.tsv", check.names
= F, sep = "\t", header = F, row.names = 1)
temp_ASV[[4]] <- read.csv("ASV_TABLE_filtered_choped_barcode04.fastq.gz.fasta.blast.tab.tsv", check.names
= F, sep = "\t", header = F, row.names = 1)
temp_ASV[[5]] <- read.csv("ASV_TABLE_filtered_choped_barcode05.fastq.gz.fasta.blast.tab.tsv", check.names
= F, sep = "\t", header = F, row.names = 1)
temp_ASV[[6]] <- read.csv("ASV_TABLE_filtered_choped_barcode06.fastq.gz.fasta.blast.tab.tsv", check.names
= F, sep = "\t", header = F, row.names = 1)
temp_ASV[[7]] <- read.csv("ASV_TABLE_filtered_choped_barcode07.fastq.gz.fasta.blast.tab.tsv", check.names
= F, sep = "\t", header = F, row.names = 1)
temp_ASV[[8]] <- read.csv("ASV_TABLE_filtered_choped_barcode08.fastq.gz.fasta.blast.tab.tsv", check.names
= F, sep = "\t", header = F, row.names = 1)
temp_ASV[[9]] <- read.csv("ASV_TABLE_filtered_choped_barcode09.fastq.gz.fasta.blast.tab.tsv", check.names
= F, sep = "\t", header = F, row.names = 1)
temp_ASV[[10]] <- read.csv("ASV_TABLE_filtered_choped_barcode10.fastq.gz.fasta.blast.tab.tsv", check.names
= F, sep = "\t", header = F, row.names = 1)
temp_ASV[[11]] <- read.csv("ASV_TABLE_filtered_choped_barcode11.fastq.gz.fasta.blast.tab.tsv", check.names
= F, sep = "\t", header = F, row.names = 1)
temp_ASV[[12]] <- read.csv("ASV_TABLE_filtered_choped_barcode12.fastq.gz.fasta.blast.tab.tsv", check.names
= F, sep = "\t", header = F, row.names = 1)

names(temp_ASV) <- barcodes

#The following function will deal with empty barcodes (like blanks)
for(i in 1:length(temp_ASV)) {
```

```
  if (lapply(temp_ASV[i], function(df) nrow(df)) == 0) {
    temp_ASV[i] <- list(data.frame(V1 = rep(0, times = 1)))
    rownames(temp_ASV[[i]]) <- rownames(temp_ASV[[i-1]])[nrow(temp_ASV[[i-1]])]
  }
}

for (i in 1:length(temp_ASV)){
  if (rownames(temp_ASV[[i]])[1] == "*"){
    rn <- rownames(temp_ASV[[i]])[-1]
    temp_ASV[[i]] <- data.frame(temp_ASV[[i]][-1,], check.rows = F, check.names = F)
    rownames(temp_ASV[[i]]) <- rn
  }
}

for (i in 1:length(temp_ASV)) colnames(temp_ASV[[i]]) <- barcodes[i]

names(temp_ASV) <- barcodes[1:length(temp_ASV)]

##ASV Taxonomy ----
#Individual taxonomy tables loading
temp_TAX = list.files(path = paste0("."), pattern="*Taxonomy.txt")
for (i in 1:length(temp_TAX)) assign(temp_TAX[i], data.frame(read.csv2(file = paste("./",temp_TAX[i], sep
= ""), sep = ";", header = F, check.names = F, fill = TRUE,
                                                                      col.names = c("Kingdom", "Phylum",
"Class", "Order", "Family", "Genus",
                                                                                 "Species", "other1",
"other2", "other3", "other4", "other5", "other6",
                                                                                 "others7", "others8",
"others9", "others10", "others11", "others12", "others13", "others14", "others15", "others16", "others17",
"others19"))))
#The reason we allow so many fields is that Chloroplasts and Mitochondria (mainly), have non-coherent
taxonomy sizes compared to Bacterial full taxonomy.
#To avoid any "Out of field" errors or alike, we allow them to exist in a first time

temp_TAX <- mget(temp_TAX)

for(i in 1: length(temp_TAX)){
  if (lapply(temp_TAX[i], function(df) nrow(df)) != 0) {#This step allows to correct for Kingdom, which is
currently merged with SILVA ID
    rownames(temp_TAX[[i]]) <- sapply(strsplit(temp_TAX[[i]][,1], split = " "), "[[", 1)
    temp_TAX[[i]][,1] <- sapply(strsplit(temp_TAX[[i]][,1], split = " "), "[", 2)
    # if(file.exists(paste0(OUTPWD,"/Results/Unknown_clusters/unknown_clusters.tsv"))){
    #   temp_TAX[[i]] <- rbind(temp_TAX[[i]], U_TAX)
    # }
  }
}

for(i in 1: length(temp_TAX)) {
  if (nrow(temp_TAX[[i]]) == 0) {
    temp_TAX[[i]][1,] <- temp_TAX[[i-1]][1,]
    rownames(temp_TAX[[i]]) <- rownames(temp_ASV[[i-1]])[length(rownames(temp_ASV[[i-1]]))]
  }
}

temp_phyloseq <- barcodes

##Phyloseq objects ----

physeq_list <- list()
```

```
for (i in 1:length(temp_ASV)) {
  # Create the phyloseq object
  physeq_object <- phyloseq::phyloseq(phyloseq::otu_table(temp_ASV[[i]], taxa_are_rows = TRUE),
                                      phyloseq::tax_table(as.matrix(temp_TAX[[1]])),
                                      phyloseq::sample_data(metadata))

  # Get the name from the barcodes vector
  barcode_name <- barcodes[i]
  # Assign the phyloseq object to the list with the dynamic name
  physeq_list[[barcode_name]] <- physeq_object
}


i<-1 #Reset the incrementation
#Initialize the phyloseq object
NanoASV <- phyloseq::merge_phyloseq(physeq_list[[i]], physeq_list[[i + 1]])

# If more than 2 samples, then, adding them all together
if (length(physeq_list) > 2) {
  for (i in 3:length(physeq_list)) {
    NanoASV <- phyloseq::merge_phyloseq(NanoASV, physeq_list[[i]])
  }
}

Nygaard <- NanoASV
##Dataset cleaning ----
#Delete bad entries such as Eukaryota, Cyanobacteria and Archea if any
Nygaard <- phyloseq::subset_taxa(Nygaard, Kingdom != "Eukaryota")
Nygaard <- phyloseq::subset_taxa(Nygaard, Family != "Mitochondria")
Nygaard <- phyloseq::subset_taxa(Nygaard, Order != "Chloroplast")
##Taxonomy cleaning ----
#After those functions, there is no more taxa with mixed up names so we can remove supp fields of taxa
table
tax_table(Nygaard) <- phyloseq::tax_table(Nygaard)[,1:7]

#Phyloseq export ----
print("Exporting results")
save(Nygaard, file = paste0("./Nygaard.rdata"))

#CSV export -----
#Create the TAXOTU file, encompassing taxonomy and abundance
TAXOTU <- data.frame(Nygaard@tax_table, Nygaard@otu_table)
#Exporting the file for people needing this format as well
write.csv(TAXOTU, file = "./Taxonomy-Abundance_table.csv")
```

Pipeline comparisons

Pipeline_comparisons.r

```
library(vegan)
library(phyloseq)
library(ggpubr)

setwd("~/Software_dev/Benchmark_Nygaard/")

load("Nygaard_pipeline_analysis/Nygaard.rdata")

load("SituSeq_Nygaard_dataset/SituSeq_Nygaard.rdata")
SituSeq <- SituSeq.Nygaard

load("NanoASV_Nygaard_dataset/NanoASV_output/nanoasv.out/Results/Rdata/NanoASV.rdata")


sample_names(NanoASV) <- paste0(sample_names(NanoASV), "_NanoASV")
sample_names(Nygaard) <- paste0(sample_names(Nygaard), "_Nygaard")
sample_names(SituSeq) <- paste0(sample_names(SituSeq), "_SituSeq")


MOCK.phy <- merge_phyloseq(NanoASV, SituSeq, Nygaard)


MOCK.phy@sam_data$Pipeline <- sub("^barcode\\d+_", "", sample_names(MOCK.phy))




#OTUs table extraction
OTU <- data.frame(MOCK.phy@otu_table)
metadata <- data.frame(MOCK.phy@sam_data)
#Richness and shannon table creation
rich <- data.frame(apply(OTU>0,2,sum))

Shan <- data.frame(diversity(t(OTU), index="shannon"))


#Synthesis Richness and Diversity
alpha_tab <- data.frame( "richness" = rich,
                         "shannon" = Shan)
colnames(alpha_tab) <- c("richness", "shannon")

alpha_tab <- cbind(metadata, alpha_tab)




rich_plot <- ggplot(data = alpha_tab, aes(x = ena_accession, y = richness)) +
  geom_col(aes(fill = ena_accession)) + theme_bw() +
  facet_wrap(~ Pipeline) +
  theme(axis.title.x = element_text(size = 14),
        axis.title.y = element_text(size = 14),
        axis.text.x = element_text(angle=45, colour = "black", vjust=1, hjust = 1, size=5),
        legend.position = "none") +
  ylab("ASV-level Numerical Richness") + xlab("Nygaard samples ID") +
  labs(title = "Nygaard dataset bacterial populations Numerical Richness ",
       caption = date())
```

```
shannon_plot <- ggplot(data = alpha_tab, aes(x = ena_accession , y = shannon)) +
  geom_col(aes(fill = ena_accession)) + theme_bw() +
  facet_wrap(~ Pipeline) +
  theme(axis.title.x = element_text(size = 14),
        axis.title.y = element_text(size = 14),
        axis.text.x = element_text(angle=45, colour = "black", vjust=1, hjust = 1, size=5),
        legend.position = "right") +
  ylab("ASV-level Shannon Index") + xlab("Nygaard samples ID") +
  labs(title = "Nygaard dataset bacterial populations Shannon Index ",
       caption = date())


pdf("Figs/Pipelines_Alpha_div.pdf", he = 8, wi = 12)
ggarrange(rich_plot, shannon_plot)
dev.off()


alpha_tab_NanoASV <- alpha_tab[alpha_tab$Pipeline == "NanoASV",]
alpha_tab_Nygaard <- alpha_tab[alpha_tab$Pipeline == "Nygaard",]
alpha_tab_SituSeq <- alpha_tab[alpha_tab$Pipeline == "SituSeq",]

mod <- lm(alpha_tab_NanoASV$richness ~ alpha_tab_Nygaard$richness)
summary(mod)
plot(alpha_tab_NanoASV$richness ~ alpha_tab_Nygaard$richness)

mod <- lm(alpha_tab_NanoASV$shannon ~ alpha_tab_Nygaard$shannon)
summary(mod)
plot(alpha_tab_NanoASV$shannon ~ alpha_tab_Nygaard$shannon)

mod <- lm(alpha_tab_NanoASV$richness ~ alpha_tab_SituSeq$richness)
summary(mod)
plot(alpha_tab_NanoASV$richness ~ alpha_tab_SituSeq$richness)

mod <- lm(alpha_tab_NanoASV$shannon ~ alpha_tab_SituSeq$shannon)
summary(mod)
plot(alpha_tab_NanoASV$shannon ~ alpha_tab_SituSeq$shannon)

mod <- lm(alpha_tab_Nygaard$richness ~ alpha_tab_SituSeq$richness)
summary(mod)
plot(alpha_tab_Nygaard$richness ~ alpha_tab_SituSeq$richness)

mod <- lm(alpha_tab_Nygaard$shannon ~ alpha_tab_SituSeq$shannon)
summary(mod)
plot(alpha_tab_Nygaard$shannon ~ alpha_tab_SituSeq$shannon)


library(corrplot)
richness_df <- data.frame(NanoASV_richness = alpha_tab_NanoASV$richness,
                          Nygaard_richness = alpha_tab_Nygaard$richness,
                          SituSeq_richness = alpha_tab_SituSeq$richness)

cor(richness_df)

shannon_df <- data.frame(NanoASV_shannon = alpha_tab_NanoASV$shannon,
                         Nygaard_shannon = alpha_tab_Nygaard$shannon,
                         SituSeq_shannon = alpha_tab_SituSeq$shannon)

cor(shannon_df)
```

```
MOCK.genus <- tax_glom(MOCK.phy, taxrank = "Genus")
MOCK.phylum <- tax_glom(MOCK.phy, taxrank = "Phylum")

couleurs_genus <- c("#FF0000FF","#FF9900FF","#FFCC00FF","#00FF00FF","#6699FFFF",
                    "#CC33FFFF","#99991EFF","#FF00CCFF", "#999999FF","#CC0000FF",
                    "#FFCCCCFF","#FFFF00FF","#CCFF00FF","#358000FF","#0000CCFF",
                    "#99CCFFFF","#00FFFFFF","#CCFFFFFF","#9900CCFF","#CC99FFFF",
                    "#996600FF","#79CC3DFF","#CCCCCCFF","#79CC3DFF","#CCCC99FF","black")

#Genus taxonomy ----

Genus.norm <- transform_sample_counts(MOCK.genus, function(x) x/sum(x))
Genus.melted <- psmelt(Genus.norm)
sub_Genus.melted <- Genus.melted
sub_Genus.melted[sub_Genus.melted$Abundance<0.04,11:16] <- "Z_Others"

#pdf("Figs/Pipelines_Genus_composition.pdf", he = 6, wi = 12)
jpeg("Figs/Pipelines_Genus_composition.jpg", he = 6, wi = 12, units = "in", res = 1200)
ggplot(sub_Genus.melted, aes(x = Sample, y = Abundance, fill = Genus), color = "black") +
  theme_bw() +

  geom_bar(stat = "identity", color = "black", size = 0.15, width = 0.85) +
  scale_fill_manual(values = couleurs_genus) +
  ylab("Relative Abundance") +
  scale_y_continuous(expand = c(0,0)) + #
  theme(axis.title.x = element_blank(),
        axis.text.x = element_text(angle = 45, hjust = 1, vjust = 1, size = 10),
        axis.ticks.x = element_blank(),
        panel.background = element_blank(),
        panel.grid.major = element_blank(),
        panel.grid.minor = element_blank(),
        legend.position = "right",
        plot.margin = margin(t = 10, r = 50, b = 10, l = 50, unit = "pt")) +
  labs(title = "Nygaard dataset samples Genus-level composition")
#subtitle = "",
#caption = date())
dev.off()

#Phylum taxonomy ----

Phylum.norm <- transform_sample_counts(MOCK.phylum, function(x) x/sum(x))
Phylum.melted <- psmelt(Phylum.norm)
sub_Phylum.melted <- Phylum.melted
sub_Phylum.melted[sub_Phylum.melted$Abundance<0.04,7:8] <- "Z_Others"

pdf("Figs/Pipelines_Genus_composition.pdf", he = 6, wi = 12)
#jpeg("Figs/Pipelines_Phylum_composition.jpg", he = 6, wi = 12, units = "in", res = 1200)
ggplot(sub_Phylum.melted, aes(x = Sample, y = Abundance, fill = Phylum), color = "black") +
  theme_bw() +

  geom_bar(stat = "identity", color = "black", size = 0.15, width = 0.85) +
  scale_fill_manual(values = c(couleurs_genus[1:6], "black")) +
  ylab("Relative Abundance") +
  scale_y_continuous(expand = c(0,0)) + #
  theme(axis.title.x = element_blank(),
        axis.text.x = element_text(angle = 45, hjust = 1, vjust = 1, size = 10),
```

```
        axis.ticks.x = element_blank(),
        panel.background = element_blank(),
        panel.grid.major = element_blank(),
        panel.grid.minor = element_blank(),
        legend.position = "right",
        plot.margin = margin(t = 10, r = 50, b = 10, l = 50, unit = "pt")) +
  labs(title = "Nygaard dataset samples Genus-level composition")
#subtitle = "",
#caption = date())
dev.off()


# Beta div ----
library(ape)


dist.bray <- vegdist(t(MOCK.genus@otu_table), method = "bray")
pcoa.sub <- pcoa(dist.bray)
beta_tab <- cbind(pcoa.sub$vectors[,1:2], MOCK.genus@sam_data)


#Global Beta diversity ----
pdf("Figs/Beta_div.pdf", he = 8, wi =12)
ggplot(beta_tab) + theme_bw() +
  geom_point(aes(x = Axis.1, y = Axis.2,
                 color = Pipeline),
             size = 5) +
  geom_text(data = beta_tab, aes(x = Axis.1, y = Axis.2), label = sub("_.*$", "", rownames(beta_tab)),
size = 4) +
  geom_hline(yintercept = 0, lty = 3) +
  geom_vline(xintercept = 0, lty = 3) +
  xlab(paste("PCo1 (", round(pcoa.sub$values$Relative_eig[1]*100, 1), "%)")) +
  ylab(paste("PCo2 (", round(pcoa.sub$values$Relative_eig[2]*100, 1), "%)")) +
  labs(title = "Nygaard dataset Bacterial population PCoA \nBray-Curtis distance matrix",
       #subtitle = "Plot of length by dose",
       caption = date(),
       color = "Pipeline",
       shape = "Sample")
dev.off()

#No SituSeq BetDiv ----

NoSituSeq <- subset_samples(MOCK.genus, MOCK.genus@sam_data$Pipeline != "SituSeq")

dist.bray <- vegdist(t(NoSituSeq@otu_table), method = "bray")
pcoa.sub <- pcoa(dist.bray)
beta_tab <- cbind(pcoa.sub$vectors[,1:2], NoSituSeq@sam_data)

pdf("Figs/Beta_div_No_SituSeq.pdf", he = 8, wi =12)
ggplot(beta_tab) + theme_bw() +
  geom_point(aes(x = Axis.1, y = Axis.2,
                 color = Pipeline),
             size = 5) +
  geom_text(data = beta_tab, aes(x = Axis.1, y = Axis.2), label = sub("_.*$", "", rownames(beta_tab)),
size = 4) +
  geom_hline(yintercept = 0, lty = 3) +
  geom_vline(xintercept = 0, lty = 3) +
  xlab(paste("PCo1 (", round(pcoa.sub$values$Relative_eig[1]*100, 1), "%)")) +
  ylab(paste("PCo2 (", round(pcoa.sub$values$Relative_eig[2]*100, 1), "%)")) +
```

```
  labs(title = "Nygaard dataset Bacterial population PCoA \nBray-Curtis distance matrix \nNo SituSeq",
       caption = date(),
       color = "Pipeline",
       shape = "Sample")
dev.off()


#Individual Beta div ----


NanoASV.beta <- subset_samples(MOCK.genus, MOCK.genus@sam_data$Pipeline == "NanoASV")

dist.bray <- vegdist(t(NanoASV.beta@otu_table), method = "bray")
dist.NanoASV <- dist.bray
pcoa.sub <- pcoa(dist.bray)
beta_tab <- cbind(pcoa.sub$vectors[,1:2], NanoASV.beta@sam_data)

pdf("Figs/NanoASV_Beta_div.pdf", he = 8, wi =12)
ggplot(beta_tab) + theme_bw() +
  geom_point(aes(x = Axis.1, y = Axis.2),
                 color = "red",
             size = 5) +
  geom_text(data = beta_tab, aes(x = Axis.1, y = Axis.2), label = sub("_.*$", "", rownames(beta_tab)),
size = 4) +
  geom_hline(yintercept = 0, lty = 3) +
  geom_vline(xintercept = 0, lty = 3) +
  xlab(paste("PCo1 (", round(pcoa.sub$values$Relative_eig[1]*100, 1), "%)")) +
  ylab(paste("PCo2 (", round(pcoa.sub$values$Relative_eig[2]*100, 1), "%)")) +
  labs(title = "Nygaard dataset Bacterial population PCoA \nBray-Curtis distance matrix \nNanoASV
workflow",
       caption = date(),
       color = "Pipeline",
       shape = "Sample")
dev.off()


Nygaard.beta <- subset_samples(MOCK.genus, MOCK.genus@sam_data$Pipeline == "Nygaard")

dist.bray <- vegdist(t(Nygaard.beta@otu_table), method = "bray")
dist.Nygaard <- dist.bray
pcoa.sub <- pcoa(dist.bray)
beta_tab <- cbind(pcoa.sub$vectors[,1:2], Nygaard.beta@sam_data)

pdf("Figs/Nygaard.beta_Beta_div.pdf", he = 8, wi =12)
ggplot(beta_tab) + theme_bw() +
  geom_point(aes(x = Axis.1, y = Axis.2),
                 color = "lightgreen",
             size = 5) +
  geom_text(data = beta_tab, aes(x = Axis.1, y = Axis.2), label = sub("_.*$", "", rownames(beta_tab)),
size = 4) +
  geom_hline(yintercept = 0, lty = 3) +
  geom_vline(xintercept = 0, lty = 3) +
  xlab(paste("PCo1 (", round(pcoa.sub$values$Relative_eig[1]*100, 1), "%)")) +
  ylab(paste("PCo2 (", round(pcoa.sub$values$Relative_eig[2]*100, 1), "%)")) +
  labs(title = "Nygaard dataset Bacterial population PCoA \nBray-Curtis distance matrix \nNygaard
pipeline",
       caption = date(),
       color = "Pipeline",
       shape = "Sample")
```

```
dev.off()


SituSeq.beta <- subset_samples(MOCK.genus, MOCK.genus@sam_data$Pipeline == "SituSeq")

dist.bray <- vegdist(t(SituSeq.beta@otu_table), method = "bray")
dist.SituSeq <- dist.bray
pcoa.sub <- pcoa(dist.bray)
beta_tab <- cbind(pcoa.sub$vectors[,1:2], SituSeq.beta@sam_data)

pdf("Figs/SituSeq.beta_Beta_div.pdf", he = 8, wi =12)
ggplot(beta_tab) + theme_bw() +
  geom_point(aes(x = Axis.1, y = Axis.2),
             color = "lightblue",
             size = 5) +
  geom_text(data = beta_tab, aes(x = Axis.1, y = Axis.2), label = sub("_.*$", "", rownames(beta_tab)),
size = 4) +
  geom_hline(yintercept = 0, lty = 3) +
  geom_vline(xintercept = 0, lty = 3) +
  xlab(paste("PCo1 (", round(pcoa.sub$values$Relative_eig[1]*100, 1), "%)")) +
  ylab(paste("PCo2 (", round(pcoa.sub$values$Relative_eig[2]*100, 1), "%)")) +
  labs(title = "Nygaard dataset Bacterial population PCoA \nBray-Curtis distance matrix \nNygaard
pipeline",
       caption = date(),
       color = "Pipeline",
       shape = "Sample")
dev.off()


#Mantel tests to check if dissimilarity matrices are alike

library(vegan)

mantel(dist.NanoASV, dist.Nygaard)
mantel(dist.NanoASV, dist.SituSeq)
mantel(dist.Nygaard, dist.SituSeq)
```
