# Supplementary material for: NanoASV: a snakemake workflow for reproducible field-based Nanopore full-length 16S metabarcoding amplicon data analysis
Source: Bioinformatics. 2025 Mar 20;41(3):btaf089. doi: 10.1093/bioinformatics/btaf089 (PMC11937976; doi:10.1093/bioinformatics/btaf089)
Supplement: btaf089_Supplementary_Data [file btaf089_supplementary_data.zip › SUPP_INFO_03_NanoASV_default_parameters_tuning.pdf]

Minimap2 model choice

Minimap2 offers several presets model as alignment paremeters. Three of them could be of use for full long reads mapping : asm5, asm10 and map-ont.

Asm5 was designed to map long reads against assemblies with a tolerance of 5%.

asm10 was designed to map long reads against assemblies with a tolerance of 10%.

map-ont was designed to map long and noise reads against reference with a tolerance around 10%.

User can tune the model with the --model option.

The chosen model has strong consequences on taxonomical affiliations and recovery rates as shown in the following figures

Those data were obtained against the SILVA 138.2 full dataset.

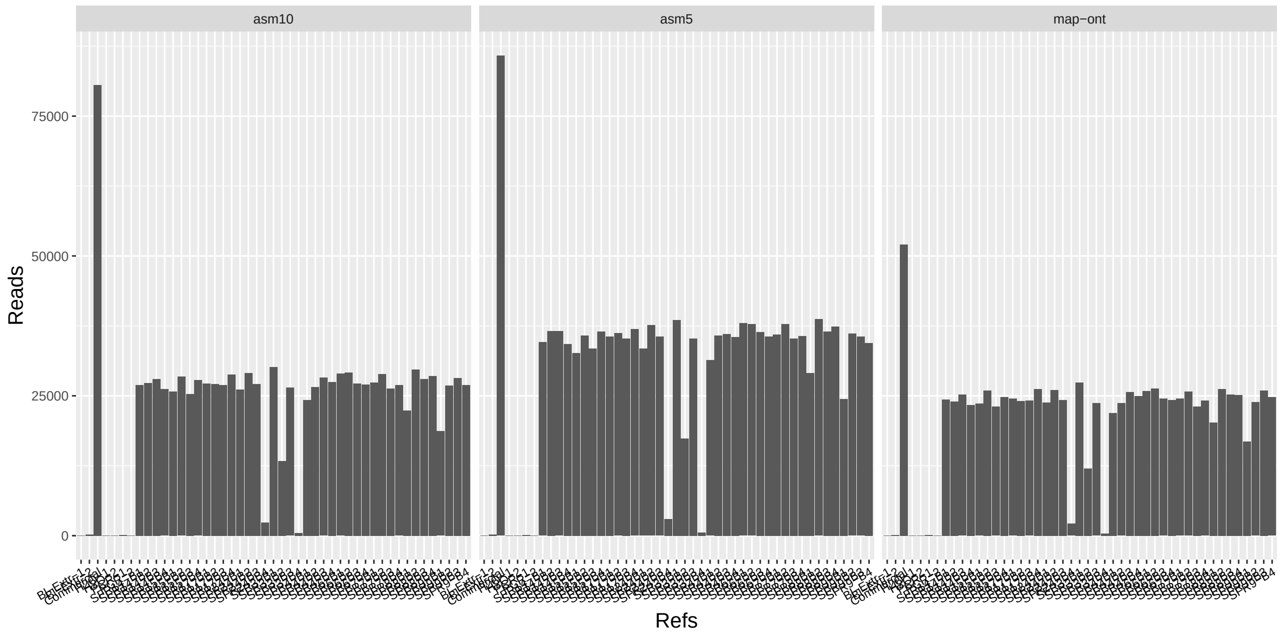

Figure 1: Different models recovery rate with no MapQ filtering

map-ont model tends to recover less reads than the other two (See Sup Fig 1). However, it’s taxonomical affiliation capacity is higher than the other two (See Sup Fig 2)

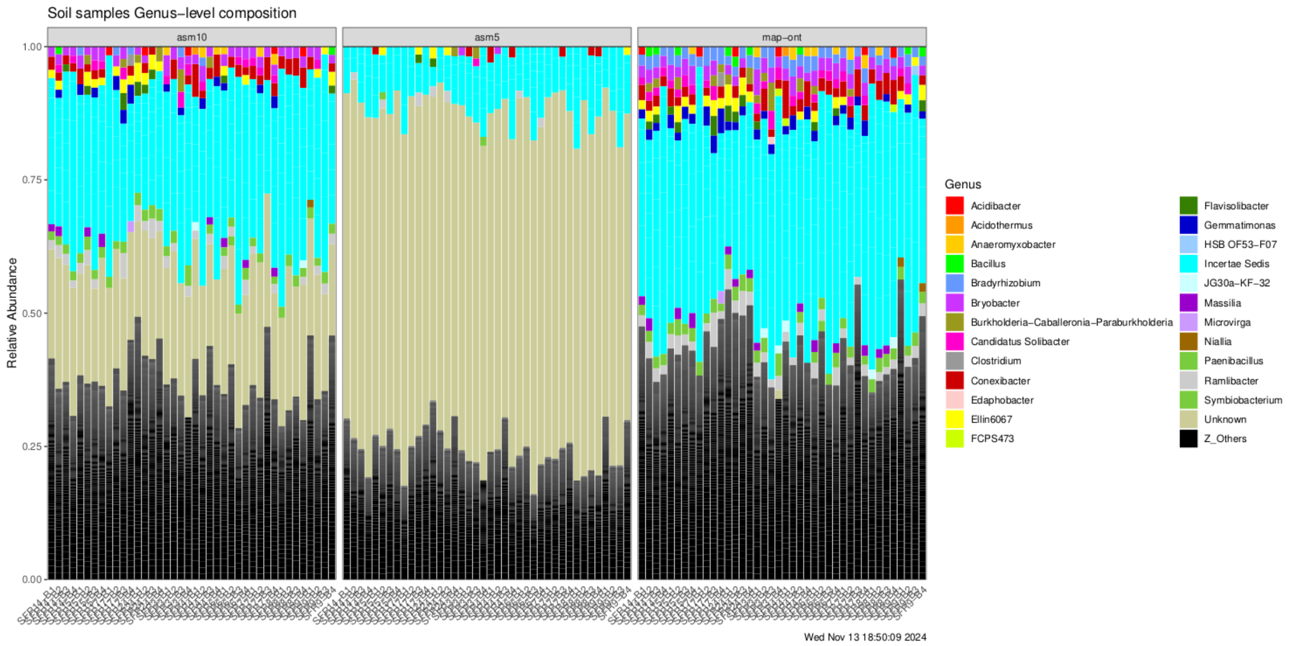

Figure 2: Affiliations taxonomical profile at Genus level depending on minimap2 model

We observe higher proportions of unknown sequences with asm5 and asm10 models compared to map-ont. This pattern is observed at all taxonomical levels up to the phylum (See supplementary material : Composition_with_different_models.pdf)

To have a correct idea of minimap2 model efficiency, we cannot rely on highly unknown soil bacterial population. The test dataset contains mock positive controls (ZymoBIOMICS Microbial Community DNA Standard II (Log Distribution)). (See Sup Fig 3)

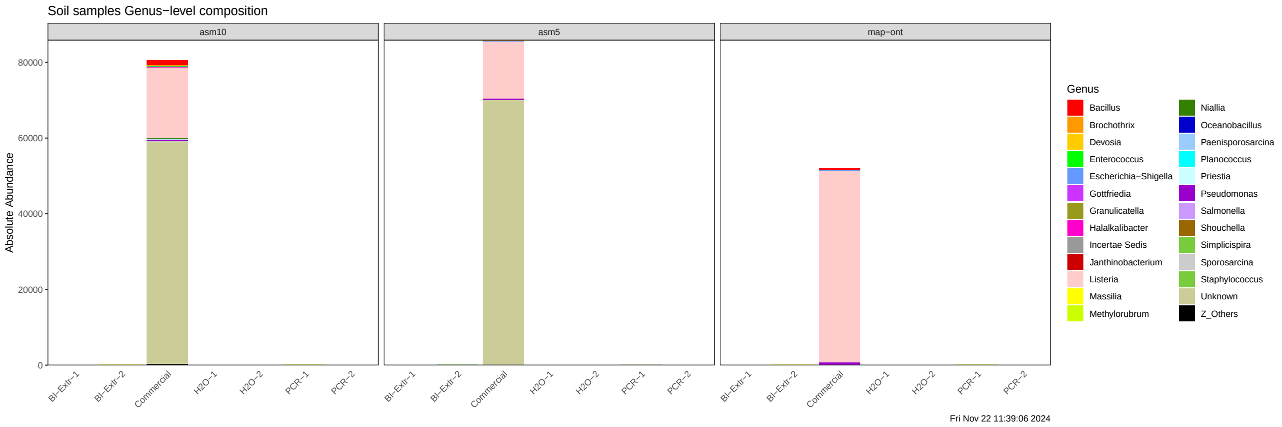

Figure 3: Sequencing controls absolute composition

The composition of the mock community is perfectly known (*Listeria monocytogenes* - 89.1%, *Pseudomonas aeruginosa* - 8.9%, *Bacillus subtilis* - 0.89%, *Saccharomyces cerevisiae* - 0.89%, *Escherichia coli* - 0.089%, *Salmonella enterica* - 0.089%, *Lactobacillus fermentum* - 0.0089%, *Enterococcus faecalis* - 0.00089%, *Cryptococcus neoformans* - 0.00089%, and *Staphylococcus aureus* – 0.000089%). The problem in Sup Fig 3 is that the mock community composition should always be resolved. We cannot tolerate 75% of unknown in perfectly known community. This results by itself justifies the use of the map-ont minimap2 model for alignment.

Diversity indices variations depending on minimap2 models

Despite a low taxonomic profile recovery rate, alpha diversity metrics are conserved between the different models (See Sup Fig 4)

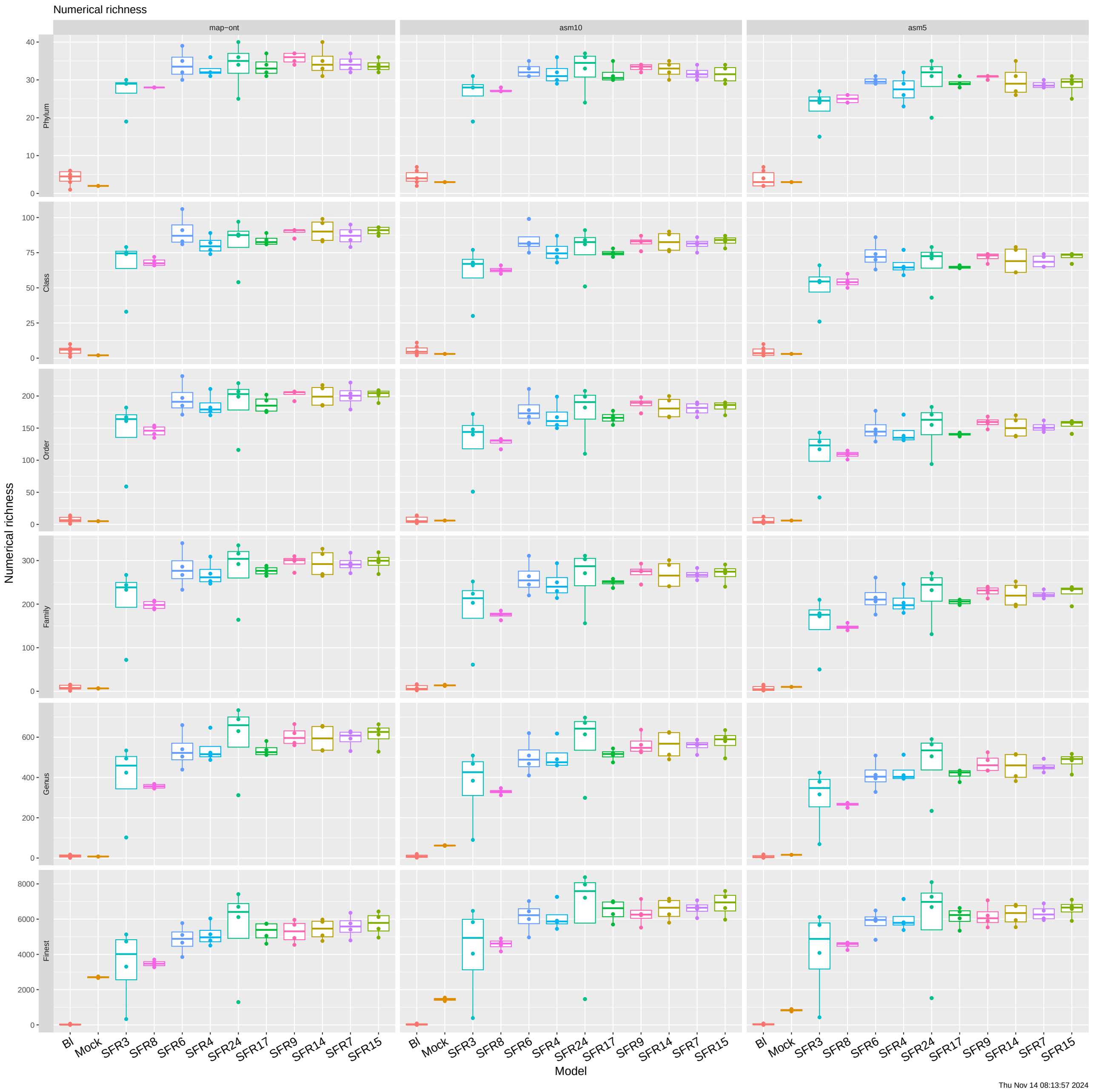

Figure 4: Numerical richness at different taxonomical levels depending on chosen minimap2 model

With Sup Fig 3, we can see that alpha diversity trends are conserved between the different minimap2 models. Taxonomical profiling will be leading in choice.

Samtools MapQ threshold value

Another important parameters is about the confidence given to an alignment.

MapQ value is not an average nucleotide identity (ANI).

MAPQ (Mapping Quality) is a metric that indicates the confidence in the alignment of a read to a reference genome. It is calculated as:

$$MAPQ = -10 * \log10(P)$$

where P is the probability that the alignment is incorrect. Higher MAPQ values indicate greater confidence. Typical MAPQ values: - 0: Low confidence or multiple mapping locations. - >30: High confidence alignment. MapQ threshold of 10 corresponds to a 90% confidence with the alignment knowing the reference composition.

MapQ calculation rely on reference composition. If lots of very similar sequences are in the reference, minimap2 will give less confidence with a specific alignment because another one could have fit as well. This does not mean that the affiliation is erroneous.

Again, we'll focus with Mock community positive controls to have an idea of MapQ threshold impact on affiliations and recovery rates

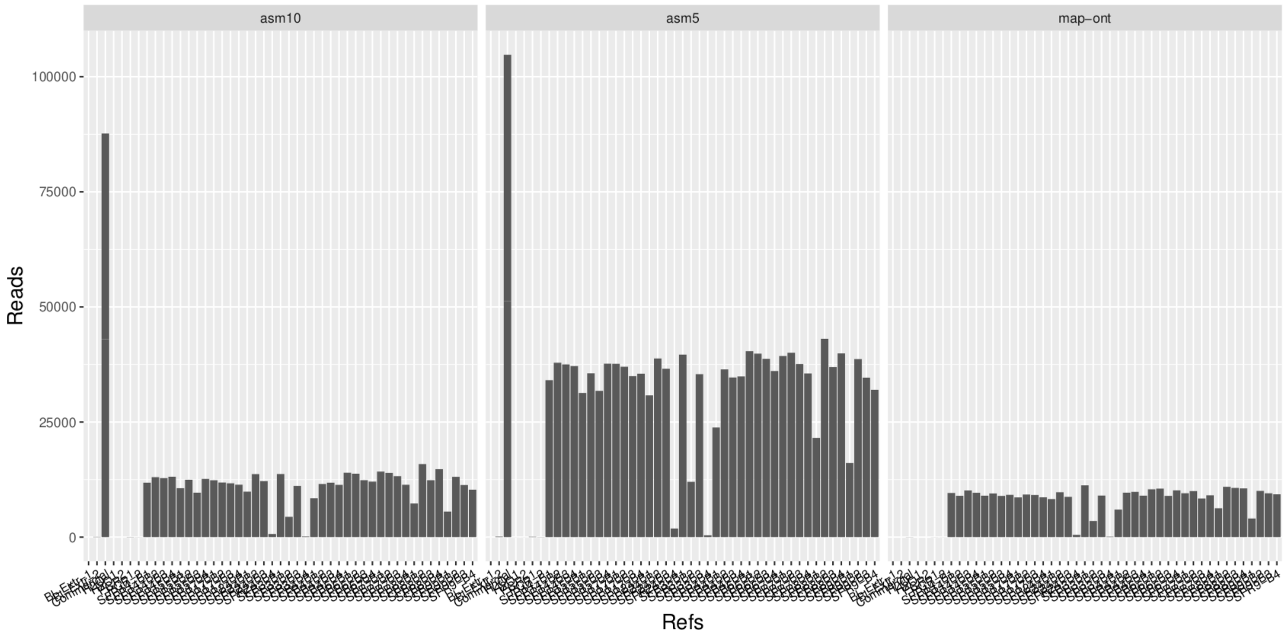

Figure 5: Q30 MapQ threshold impacts on recovery rates

We see in Sup Fig 5 that Mock recovery rates are close to zero for map-ont model. Recovery rates are higher with other models. This comes from the fact that asm5 and 10 models tend to put more reads in unknowns which are then processed by vsearch, and not samtools.

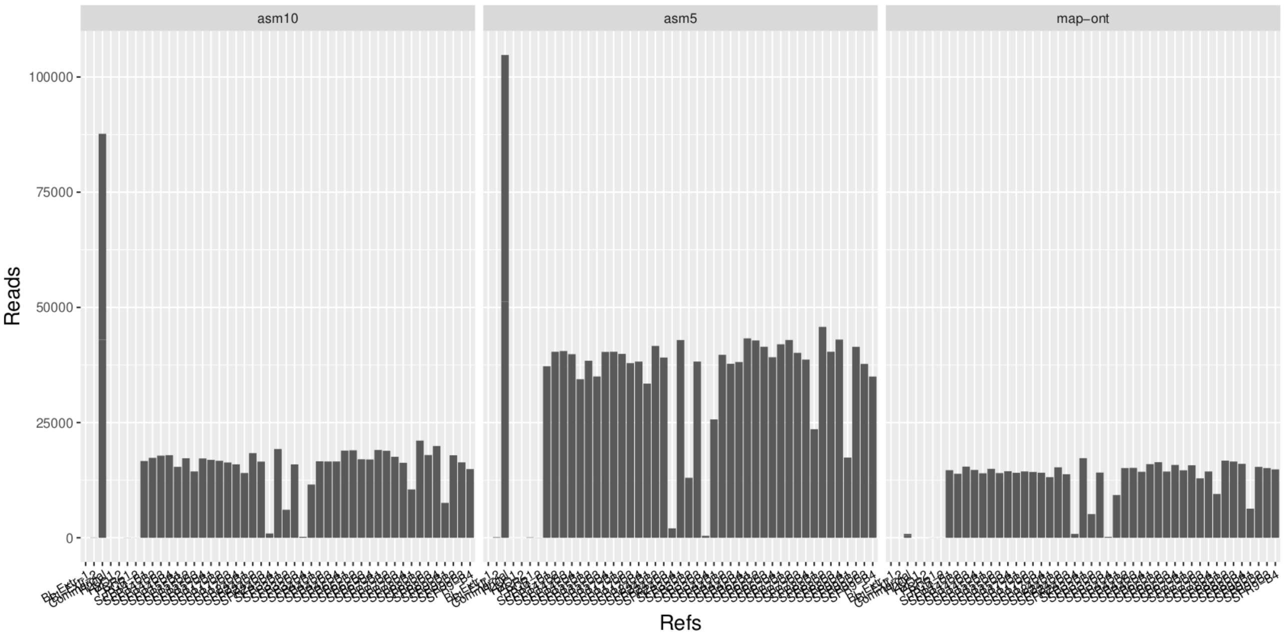

Figure 6: Q15 MapQ threshold impacts on recovery rates

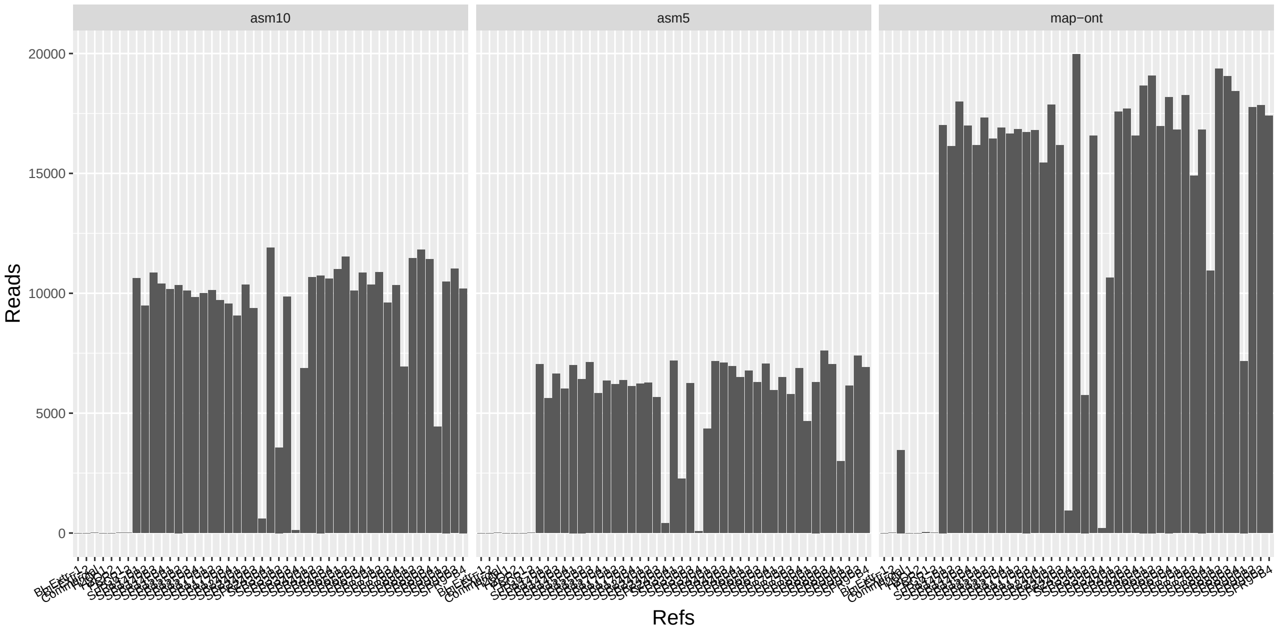

Figure 7: Q10 MapQ threshold impacts on recovery rates

With lower MapQ values (See Sup Figs 6 and 7), we observe the same phenomenon. Even MapQ threshold of 1 (See Sup Fig 8) was deleterious to positive control recovery

This led us to the conclusion that we should not set a stringent MapQ threshold by default, and set by default as 0 (See Sup Fig.9). We let the user make their own choices depending on the nature of the community they are working with and the composition of their reference dataset.

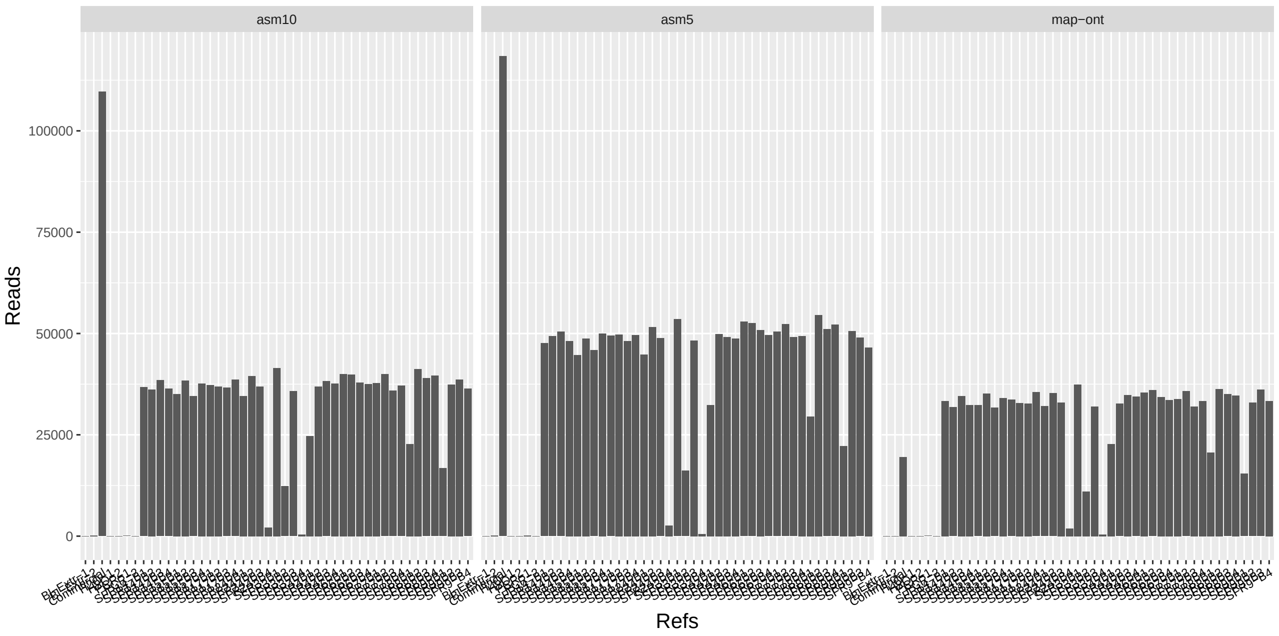

Figure 8: Q1 MapQ threshold impacts on recovery rates

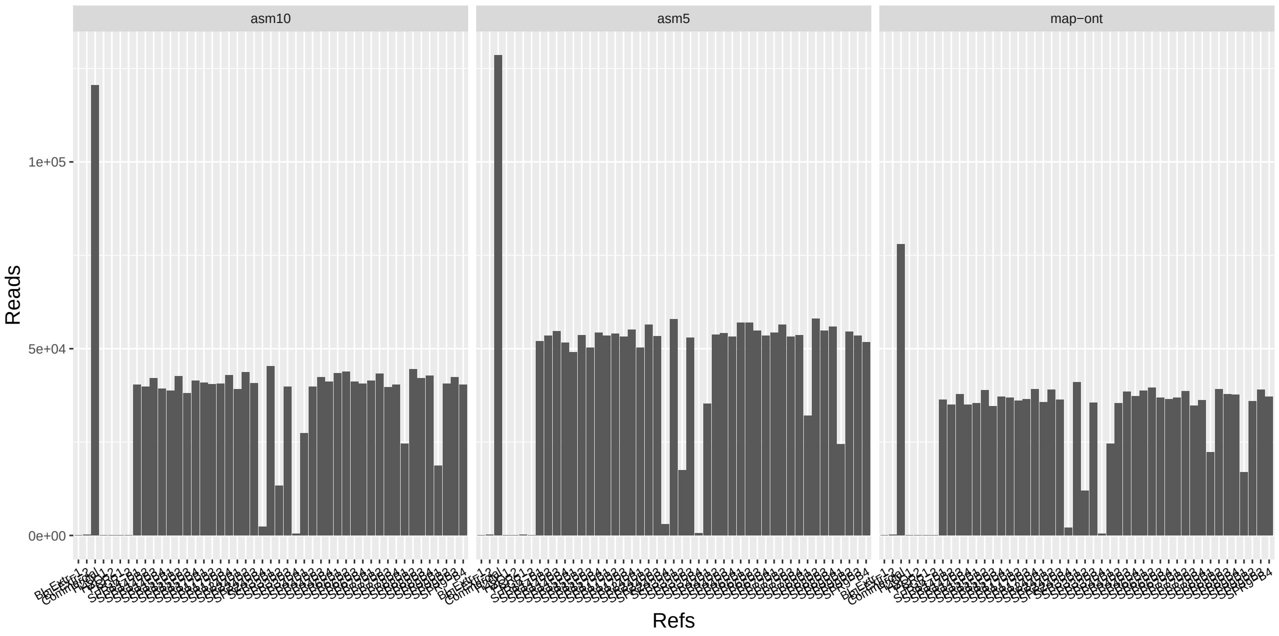

Figure 9: Q0 MapQ threshold impacts on recovery rates
