# Supplementary material for: NanoASV: a snakemake workflow for reproducible field-based Nanopore full-length 16S metabarcoding amplicon data analysis
Source: Bioinformatics. 2025 Mar 20;41(3):btaf089. doi: 10.1093/bioinformatics/btaf089 (PMC11937976; doi:10.1093/bioinformatics/btaf089)
Supplement: btaf089_Supplementary_Data [file btaf089_supplementary_data.zip › SUPP_INFO_02_Discovering_new_biological_entities_with_NanoASV.pdf]

Discovering new bacterial entities with NanoASV

One of NanoASV key feature is to allow you to detect unknown biological entities despite difficulties with ONT dereplication.

To test what is classified as *unknown*, we are investigating some of clusters consensus sequences and blast them against NCBI non-redundant database (nr)

For this exploration case, we are taking a map-ont minimap2 model and a samtools MapQ quality threshold of 15 (97% confidence in the alignment knowing the reference set), vsearch id of 0.7.

Online blast parameters : *Somewhat similar sequences (blastn) vs nr*

Cluster 79

>ad1bcb1c-1ca4-4719-b57d-c9954f80c26e_Unknown_cluster_79_clusterid=78_size=143

GTTACCTTGTTACGACTTTTCCATCTTGCGGTTTTTAACCAGAGGCCTATCGACGTGGAGCAGC
TGCACACTAATTATGAATTCGGCGCCCGTTAAGTGGCGCCCAATAGATTCGAGCGCCTAGGCT
CAAGTCTCCGCTTTCGTGGCTTCAACTGCGGTAGCGAAGATGACGTCCGCAAGTCAGACAAC
CAAAGAACCAAGCCGATCGCCAAATTCACCCGCGAGAGCAGAACGCCGCTCGATTAATAAAT
CAAGATTCTCCGGCATTGCGAAGATCGACGCATAGTAGGCACGCAGTCGACGAGTGATTTCAA
CGCGCTGCGCGCGTTCGTGAGCTCCTCGCGGGTTGTATGAGCGGTACACGGACATCGTCACG
GCGCTCTGTTCGTCCAATTAGGCAAATAACAGAGTTGCCAGTCAACTGCTGTCCGCTGATCGT
TTAAGATTTTTCGCCTTGCGGTTTTGAAAGGGGCTCCACGTGCGATCAGCTGTGCACTAACCG
TCAATTCCGCGGGCTGCTGGACGGGGATCGCAATTGATTCCGTGACAAGCGTATCATGGCAAT
GAGCTCATCAAAGTCCGCAATCGTGCGTCCAGCCATCGTTCGTTTCCCGAGCCGGGAATCCTC
TACGAGCAGCCTCGGTGCGCTTGCCTCGTGCGTGAAACAAGTGATCCGGTATGCCTCTGATAG
CGGCGCGACAGCGGACATCGCGGGAGGTCCGAGTTGTGCCCGTTAACGGACAAAAGCGCTT
TCACTAGGGTCCCTCGCCGCCTGCTCGGTTCAAAACCGTTCGAAACCCAAGATGCGCGGTGC
CGAGGTCCACTTCCTGCGGCTGGCGCGCGGCCGGGCGATAACGCGCGCAGTAGTTGGAAGCG
CACAGGAAAGAGCCGCCCTTGATGACGCGGCTTGGCGACTGGCCGGCGGTCAATGCCAGATC
GTGCAATTCGGGTCCGCTCGGATTGGCCGCATTCTCCCGCGGATGACCCGGCCGATACCAATC
GCTGGTCCACTCCCAGACATTGCCGATCATGTCGTAGAGCCCATAGCCGTTGGGTTTGAAGCA
GCCGACCGGCGCGGTTCCGGCGTAGCCGTCCTCGTTCGTGTTGTAGACGGGGAAGATGCCTT
GCCAGGTGTTGGCGATCGGCTTGCCCTCGGCATCGAACGCGCTCGACCAGTCGTCCTCGCCG
TCGCGACCGCCGCGAGCGGCAAATTCCCACTGCGCTTCGGTCGGCAGCGCATGCCCGCGCCA
ACGCGCATAAGCCATCGCATCCTCGTAGGCGATATGCGCGACCGGATGATTGTCCTTGCCCGA
AATCGAGCTGCCGGGACCAGTCGGCTCACGCCAATTGGCGCCGGGGACGTATTGCCACCATT
GCGTGAGCCGGCCTCCGCTCTGGACATTGGTAGGCGGGATGAACACCACTGAGCCATGATCA
AACT

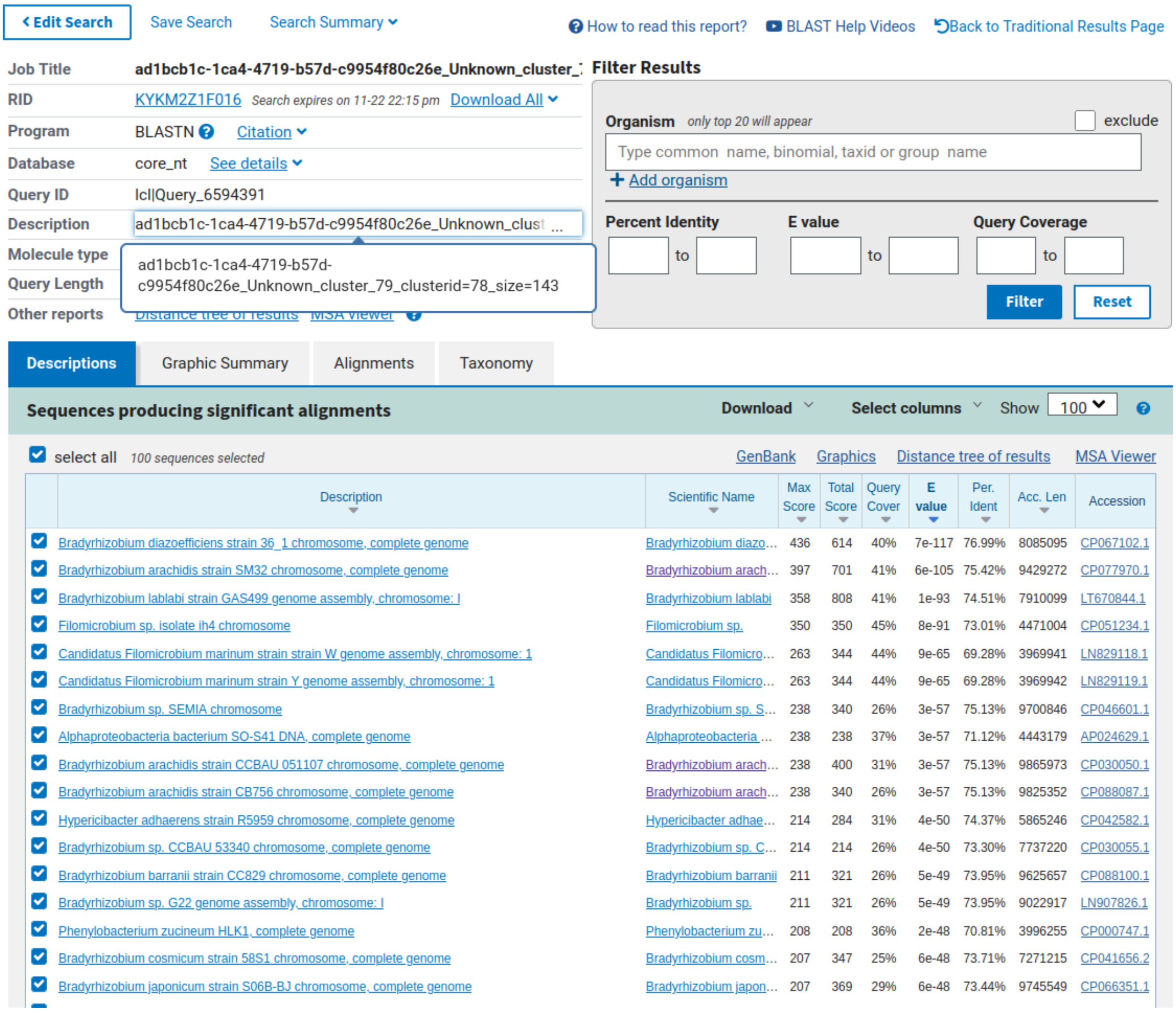

Edit Search | Save Search | Search Summary

How to read this report? | BLAST Help Videos | Back to Traditional Results Page

Job Title	ad1bcb1c-1ca4-4719-b57d-c9954f80c26e_Unknown_cluster_7
RID	KYKM2Z1F016 Search expires on 11-22 22:15 pm Download All
Program	BLASTN Citation
Database	core_nt See details
Query ID	lcl|Query_6594391
Description	ad1bcb1c-1ca4-4719-b57d-c9954f80c26e_Unknown_clust ...
Molecule type	ad1bcb1c-1ca4-4719-b57d-c9954f80c26e_Unknown_cluster_79_clusterid=78_size=143
Query Length	
Other reports	Distance tree of results MSA viewer

Filter Results

Organism only top 20 will appear — exclude

Type common name, binomial, taxid or group name

+ Add organism

Percent Identity to — **E value** to — **Query Coverage** to

Filter | Reset

Descriptions | Graphic Summary | Alignments | Taxonomy

Sequences producing significant alignments — Download — Select columns — Show 100

select all 100 sequences selected — GenBank | Graphics | Distance tree of results | MSA Viewer

Description	Scientific Name	Max Score	Total Score	Query Cover	E value	Per. Ident	Acc. Len	Accession
Bradyrhizobium diazoefficiens strain 36_1 chromosome, complete genome	Bradyrhizobium diazo...	436	614	40%	7e-117	76.99%	8085095	CP067102.1
Bradyrhizobium arachidis strain SM32 chromosome, complete genome	Bradyrhizobium arach...	397	701	41%	6e-105	75.42%	9429272	CP077970.1
Bradyrhizobium lablabi strain GAS499 genome assembly, chromosome: I	Bradyrhizobium lablabi	358	808	41%	1e-93	74.51%	7910099	LT670844.1
Filomicrobium sp. isolate ih4 chromosome	Filomicrobium sp.	350	350	45%	8e-91	73.01%	4471004	CP051234.1
Candidatus Filomicrobium marinum strain strain W genome assembly, chromosome: 1	Candidatus Filomicro...	263	344	44%	9e-65	69.28%	3969941	LN829118.1
Candidatus Filomicrobium marinum strain Y genome assembly, chromosome: 1	Candidatus Filomicro...	263	344	44%	9e-65	69.28%	3969942	LN829119.1
Bradyrhizobium sp. SEMIA chromosome	Bradyrhizobium sp. S...	238	340	26%	3e-57	75.13%	9700846	CP046601.1
Alphaproteobacteria bacterium SO-S41 DNA, complete genome	Alphaproteobacteria ...	238	238	37%	3e-57	71.12%	4443179	AP024629.1
Bradyrhizobium arachidis strain CCBAU 051107 chromosome, complete genome	Bradyrhizobium arach...	238	400	31%	3e-57	75.13%	9865973	CP030050.1
Bradyrhizobium arachidis strain CB756 chromosome, complete genome	Bradyrhizobium arach...	238	340	26%	3e-57	75.13%	9825352	CP088087.1
Hypericibacter adhaerens strain R5959 chromosome, complete genome	Hypericibacter adhae...	214	284	31%	4e-50	74.37%	5865246	CP042582.1
Bradyrhizobium sp. CCBAU 53340 chromosome, complete genome	Bradyrhizobium sp. C...	214	214	26%	4e-50	73.30%	7737220	CP030055.1
Bradyrhizobium barranii strain CC829 chromosome, complete genome	Bradyrhizobium barranii	211	321	26%	5e-49	73.95%	9625657	CP088100.1
Bradyrhizobium sp. G22 genome assembly, chromosome: I	Bradyrhizobium sp.	211	321	26%	5e-49	73.95%	9022917	LN907826.1
Phenylobacterium zucineum HLK1, complete genome	Phenylobacterium zu...	208	208	36%	2e-48	70.81%	3996255	CP000747.1
Bradyrhizobium cosmicum strain 58S1 chromosome, complete genome	Bradyrhizobium cosm...	207	347	25%	6e-48	73.71%	7271215	CP041656.2
Bradyrhizobium japonicum strain S06B-BJ chromosome, complete genome	Bradyrhizobium japon...	207	369	29%	6e-48	73.44%	9745549	CP066351.1

Figure 1: Cluster 79 blastn against nr results

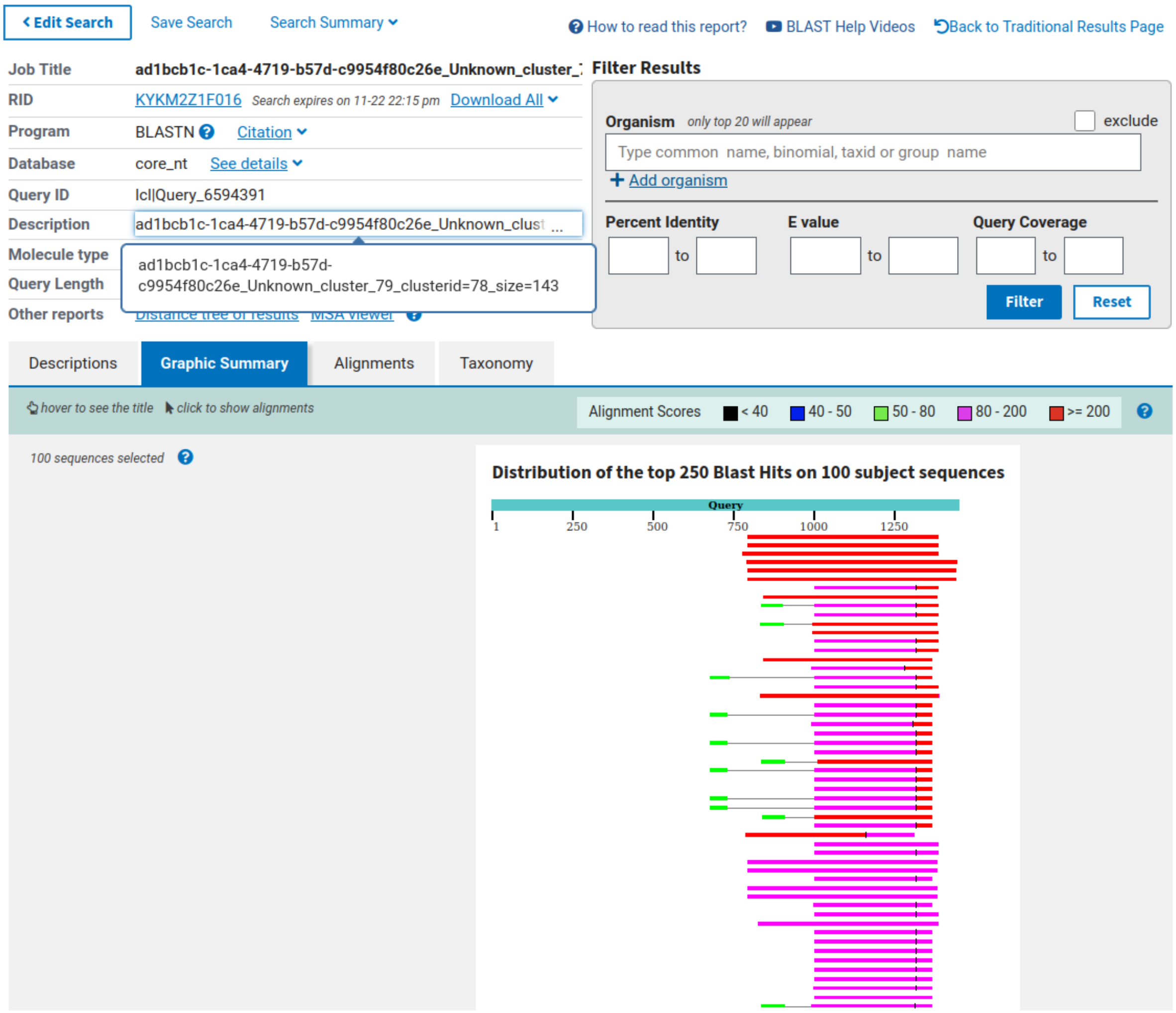

Figure 2: Cluster 79 - Graphical alignments blastn vs nr

We can see that the cluster is composed of 143 reads.

This could be chimera, because we observe correct alignment in 3' but not at all in 5'.

On the *successfully* aligned sequence, we see low ANI around 75%. close to the Phylum limit. Are we observing a molecular/sequencing artifact or an amplicon or a trace of something new ?

Lets continue digging.

Cluster 665

>ad1bcb1c-1ca4-4719-b57d-c9954f80c26e_Unknown_cluster_665_clusterid=664_size=138

AGTTTGATCATGGCTCAGTTCAATACTCTTTAGCAAGAGCACGATCGCTTCTGTGTATTTCCCC
TCCAGGATCAACATCCCACCCTGATCTTTCGTCACATCGGCAATCGCGCTCTTGTCACCAACCT
GCTCAAATAGCTCTCTACTTTGAGCATAGTAATATCGCATTTGCTCGAAGTTATATGTGAAGCC
AGCTGCCGTGCCCAGGTAAAAGAGCAACTGAGCGCGCAGCCATCTATCGTCTGCGGGAGAGA
GCAATTGCAGGCCTTGCTCGTACAAACTTTTGGCCAGTTCGTGATTGCCCTGGGCAAGAGCTG
GCCATCCACGGTATAATAATGCAGTCGCCAGGCCTCTGGGATCGTCCAATTGCCGCCAGAGCG
CGATGCTCGCTTCAGCCAGTTCTACCGCTTTGCTCTGCTCGTTTTGCAAACATACCAGGCGGG
CCACTTCGCCCAACGCTTTTGCCCTTGCTGCCAGTGCCGTCCTTCCTGCTCCTTCCACAATTGG
AACTTCCAGCACTGCATCCAGCCAGCCTCGCCCTTCAACCAGGTGGCCTTGCCATTCCCAGTA
GGCTCGAAGGGCCGCCGCCAGACGTAGAGACACCTCAACAGCAAGAAGTTCGGCTCCGGGC
ACTATCTTCAATGGCACGGCCAGCAATGAGGCGCTATCTCCAACCCGTTTCATTTCTTCCTCAT
CATTGGTCGACCTGCCCGTGGGGCTGACAGGATTGCTTGCGACCATGGTGGCTCCAGTTCTGG
CTCGTTGTAGCGACCGCTTCAAAGCCGCGCGGAAATTATCCTGCTCTGTCACCAGCCGAGCCT
GCCACATGAGTTGCTGTGGACCTCTTAATCCTATCTCAGCTGTTTCTGTCACACCCAGGTAATA
GCAGGCATGCCAATCCCGTAGACGCTCAAGCTCACCATGTGCGTTCAACCGTTCCAGCGCATA
GGCACGCAGCGTCTCCAGCAGTGTAAAGCGCACTTGCCCAGCTGTCATTGGCAGTCGTACCA
GCAAACTATTGTCAACAAGGCGCTCAAGCAGATCCAATGTGAACCCTGAAACAGTTGCGTCC
TCGTCATCCAGGTCCGTTGCAACAGCCTGCAGCATCGCTTCTGCTGCCTCAAGCGACCATCCC
CCGCTAAACACTCCCAGACGAGCAAACCACGCTTGATCGATAGGGGTGAGCAAGTCGTAACA
AGGTAAC

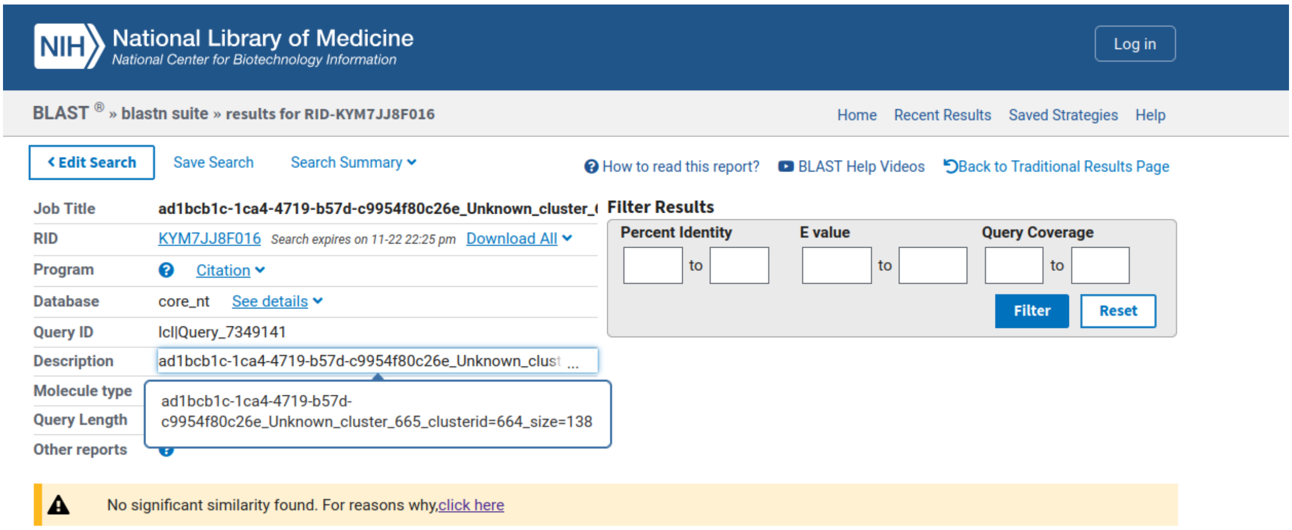

Figure 3: Cluster 665 blastn vs nr results

Difficult to say anything when we have no similarity at all.

Could be the most interesting sequence of the dataset.

Blastx against nr can give us insights

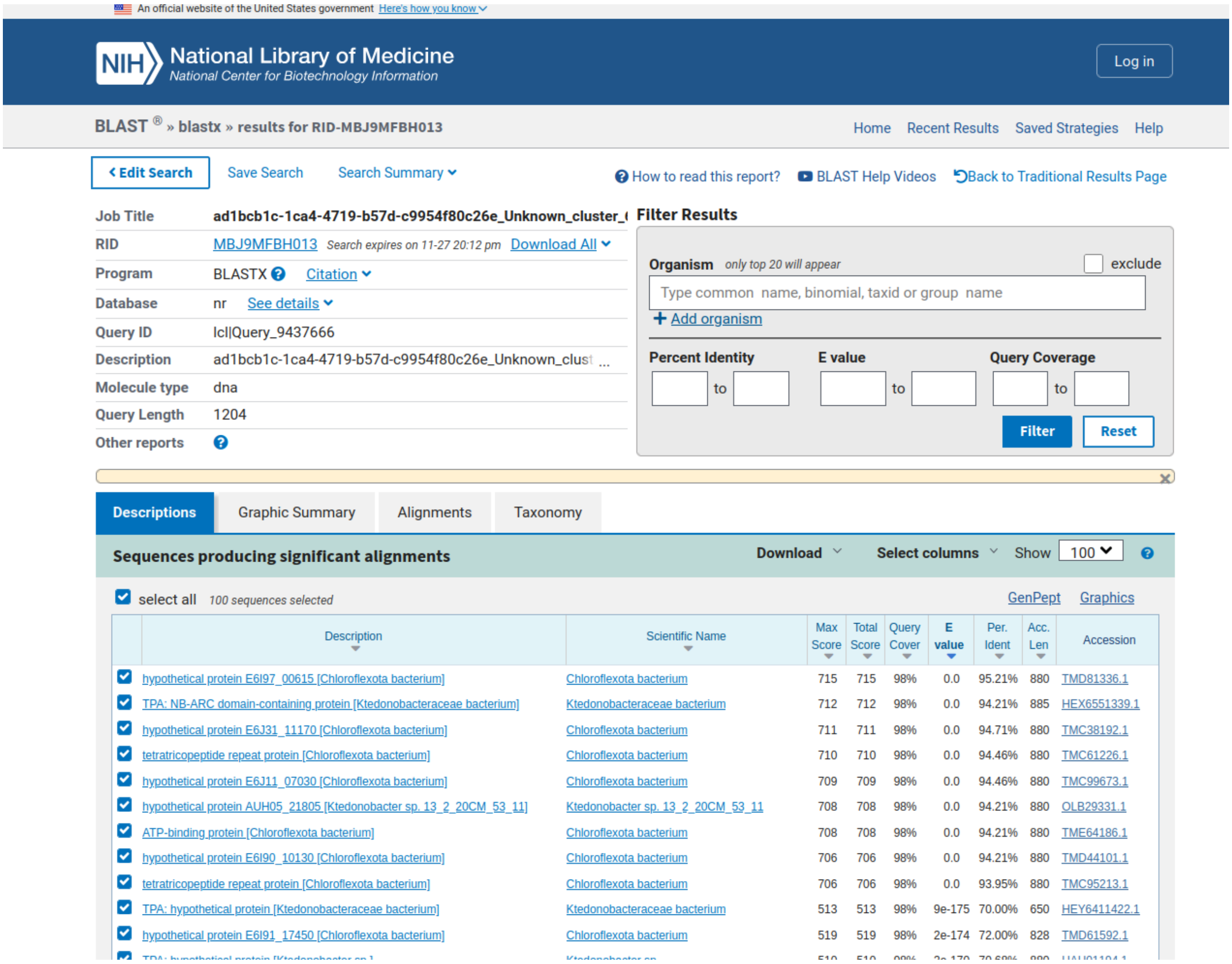

Figure 4: Cluster 665 blastx against nr results

Despite the absence of affiliation with blastn, we can see that this sequence, if coding (what should not be, because rRNA are not translated so far), has already been observed and never annotated.

What could it be ?

A very distinctive rRNA that is too divergent to be properly identified by annotation software ?

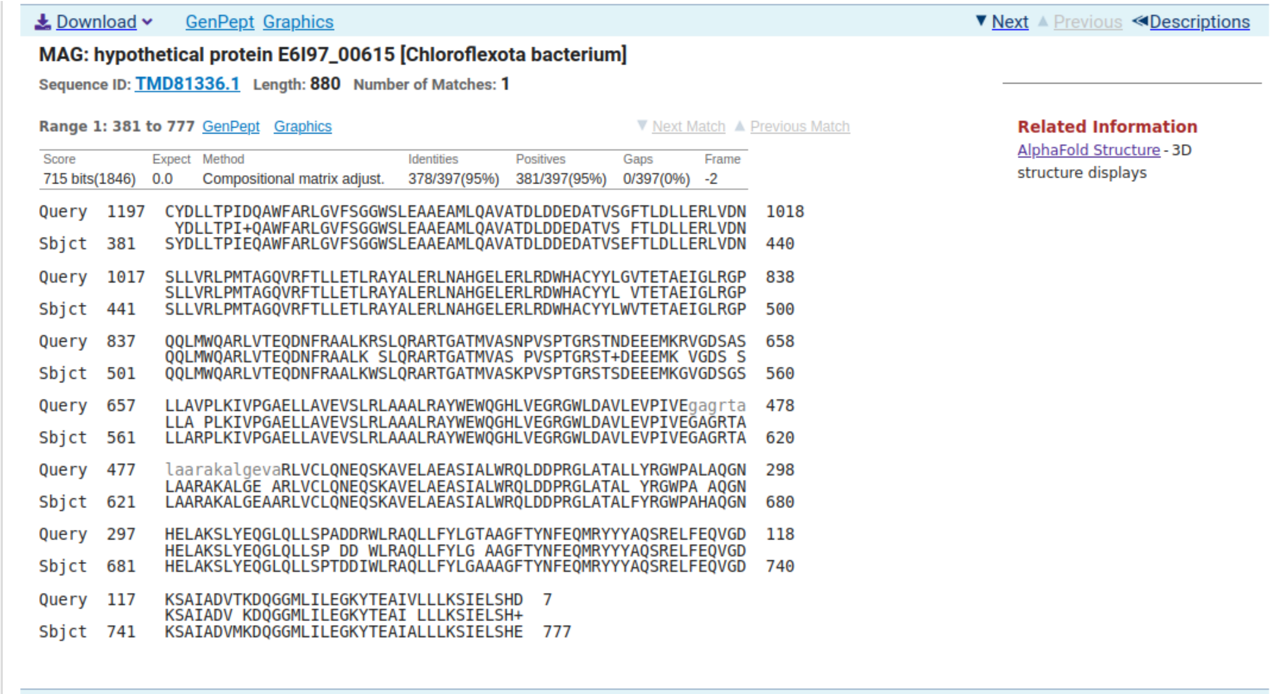

Figure 5: Predicted protein alignment with nr (blastx results)

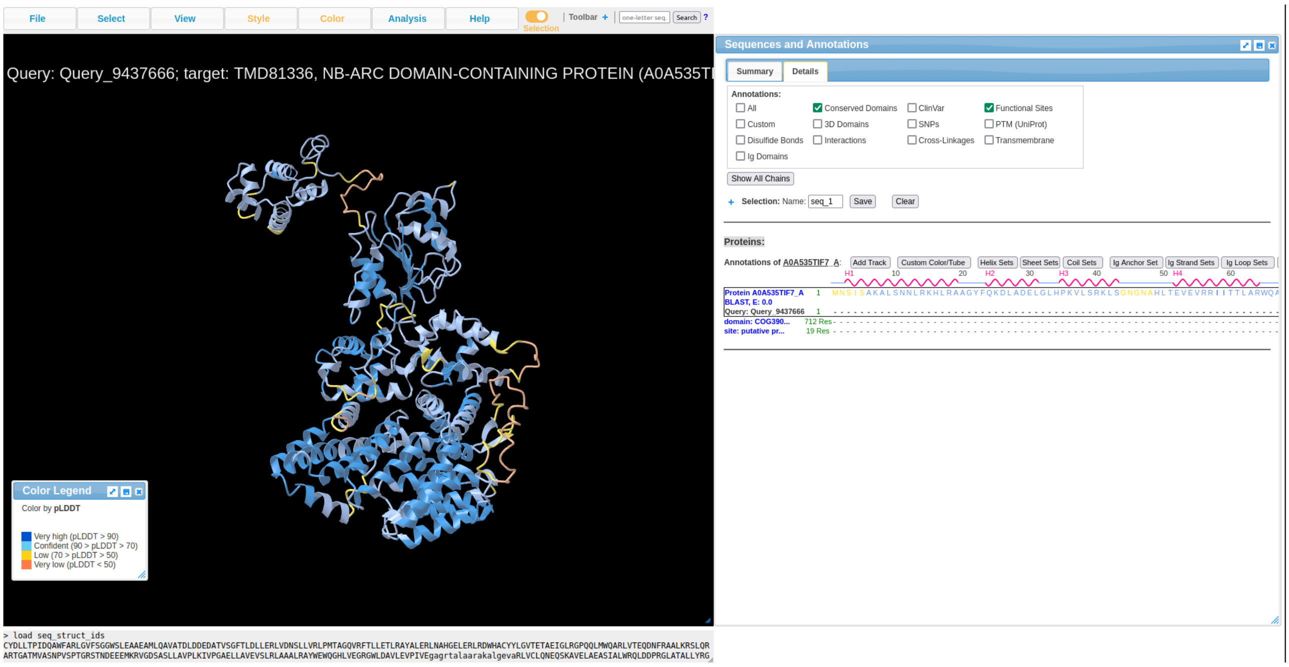

Figure 6: Alpha fold prediction for the hypothetical protein

To compare. If we take some E coli rRNA sequence and we blastx it against nr, it mapps with 50% identity to an hypothetical E coli protein. This is a weird signal

Cluster 2242

>ad1bcb1c-1ca4-4719-b57d-c9954f80c26e_Unknown_cluster_2242_clusterid=2241_size=49

TTTGATCATGGCTCAGGCGGAAGCAGCCCTTTGGTGTGGCCTAAGTAGTGTTTGTTGGACTCG
TTGTGATTGGGCTTGCGTTCTACATGTTCTCCAAGGCATACACTGCCAGGTTTAGAGCAGATC
GAATCTCACTGGTTTGAGTACACAGGTAAGGAAGTGGGTAGTCGACCTTGGTCGAATTGGATA
TGCCGCCCTTGGTGTTGTCTTTGCCATCACCGGTATCTTCCTGACCGTGGCTGCCGTTCAGCAC
AACGCCCGCGAGGCTCTAGGCCTGGATGGTACGTTGAAGGAGCTTGCGCACCAGCCGTTTGG
TTCATTGCTGCTGGGTATAATGGCTCTCGGCCCTATCGCTTATGGCGTCTATTCGTTTGTAGAGG
CTCGCTCCTGGCGCATCGGCAGATTTTAAATAGCGGGTGTTTAGAAAAACGTATGTTTTCTTTT
TTGTAATGAATATGCTATACTGTTCCCATGCTGACGTAGCTCAGTGGATAGAGCGCTTGCCTCC
GAAGCAAGAGGCCGTGGGTTCGAGTCCCGCCGTCAGCGTTATATCTCGTAGTTCTGTGCCCAA
CGGACCCCCGCACTATACTGGTTTTTCCTGTTTCCCTGTAGATGCGAGACGAAGGCTCATTTCC
CCAACAAAGCATTCATCGACATGGATCATATATACTTTCCTTATATCTGGGCTAGAGATGCCGA
AATATTCGGCATCTCCTTGATATCCCCGTTTTTGTCGTCTCCCCTCCCCACCTAAGTTATACGCA
TTGAAAAGCAAACACGTTTTATCCCTGGACAGCCGAGCCGACTTACATTGGATGCGCACCAGT
TGAGCTTTTTCACCCGAATTTGTAGAGACATAGTAGGCAAGATCATAACGGAGATGGTTGCCC
ACAGGCTCCAGGACTTCAAACCCCAATTCGATTAAACGTTGCGCTACAGCTAAGTGCGCGGCT
AACCCTTTTCTGGCTGTGTCTCCAAACACAAACACTCCTTCCTAAAACTTGACATGAGTTCTT
TACTAGATTTCATAATCCTTAGCCCATATCACTTTCTTTTCTTGGTTGTTTTTTGTCGGTTGAATG
CGCAGGTGTACTTCACCGGCATAGGGAACAATATTTATGGGAACAAGGTAAACCGTTTTTAGT
TCTGCGCAGTAAACACCGAAGTACTCAGCGTCCCCAACGTAACTACGCCTTTTACCTCGTCCT
CCTGACCTATTAAAGTTCTTAAATACGATACATCCTTTGTCAGGCTCATATCGCCCCGCTTTACA
TTGAATACGGACGAGTTCCGTAGTTTCTTCCAAATAGTATGCCAAGTCGTAACAAGGTAAC

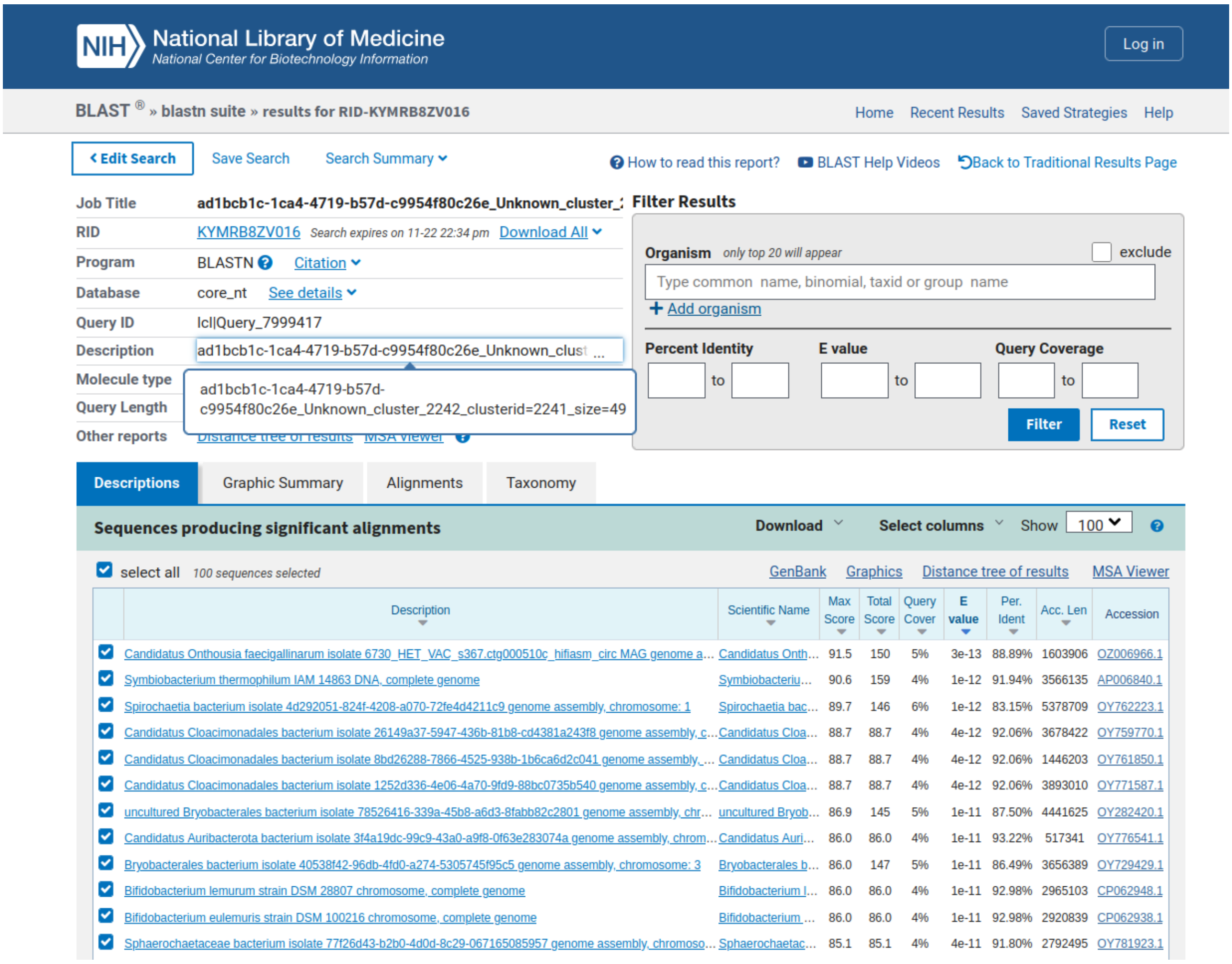

Description	Scientific Name	Max Score	Total Score	Query Cover	E value	Per. Ident	Acc. Len	Accession
Candidatus Onthousia faecigallinarum isolate 6730_HET_VAC_s367.ctg000510c_hifiasm_circ MAG genome a...	Candidatus Onth...	91.5	150	5%	3e-13	88.89%	1603906	OZ006966.1
Symbiobacterium thermophilum IAM 14863 DNA, complete genome	Symbiobacteriu...	90.6	159	4%	1e-12	91.94%	3566135	AP006840.1
Spirochaetia bacterium isolate 4d292051-824f-4208-a070-72fe4d4211c9 genome assembly, chromosome: 1	Spirochaetia bac...	89.7	146	6%	1e-12	83.15%	5378709	OY762223.1
Candidatus Cloacimonadales bacterium isolate 26149a37-5947-436b-81b8-cd4381a243f8 genome assembly, c...	Candidatus Cloa...	88.7	88.7	4%	4e-12	92.06%	3678422	OY759770.1
Candidatus Cloacimonadales bacterium isolate 8bd26288-7866-4525-938b-1b6ca6d2c041 genome assembly, ...	Candidatus Cloa...	88.7	88.7	4%	4e-12	92.06%	1446203	OY761850.1
Candidatus Cloacimonadales bacterium isolate 1252d336-4e06-4a70-9fd9-88bc0735b540 genome assembly, c...	Candidatus Cloa...	88.7	88.7	4%	4e-12	92.06%	3893010	OY771587.1
uncultured Bryobacterales bacterium isolate 78526416-339a-45b8-a6d3-8fabb82c2801 genome assembly, chr...	uncultured Bryob...	86.9	145	5%	1e-11	87.50%	4441625	OY282420.1
Candidatus Auribacterota bacterium isolate 3f4a19dc-99c9-43a0-a9f8-0f63e283074a genome assembly, chrom...	Candidatus Auri...	86.0	86.0	4%	1e-11	93.22%	517341	OY776541.1
Bryobacterales bacterium isolate 40538f42-96db-4fd0-a274-5305745f95c5 genome assembly, chromosome: 3	Bryobacterales b...	86.0	147	5%	1e-11	86.49%	3656389	OY729429.1
Bifidobacterium lemurum strain DSM 28807 chromosome, complete genome	Bifidobacterium l...	86.0	86.0	4%	1e-11	92.98%	2965103	CP062948.1
Bifidobacterium eulemuris strain DSM 100216 chromosome, complete genome	Bifidobacterium ...	86.0	86.0	4%	1e-11	92.98%	2920839	CP062938.1
Sphaerochaetaceae bacterium isolate 77f26d43-b2b0-4d0d-8c29-067165085957 genome assembly, chromoso...	Sphaerochaetac...	85.1	85.1	4%	4e-11	91.80%	2792495	OY781923.1

Figure 7: Cluster 2241 blastn vs nr results

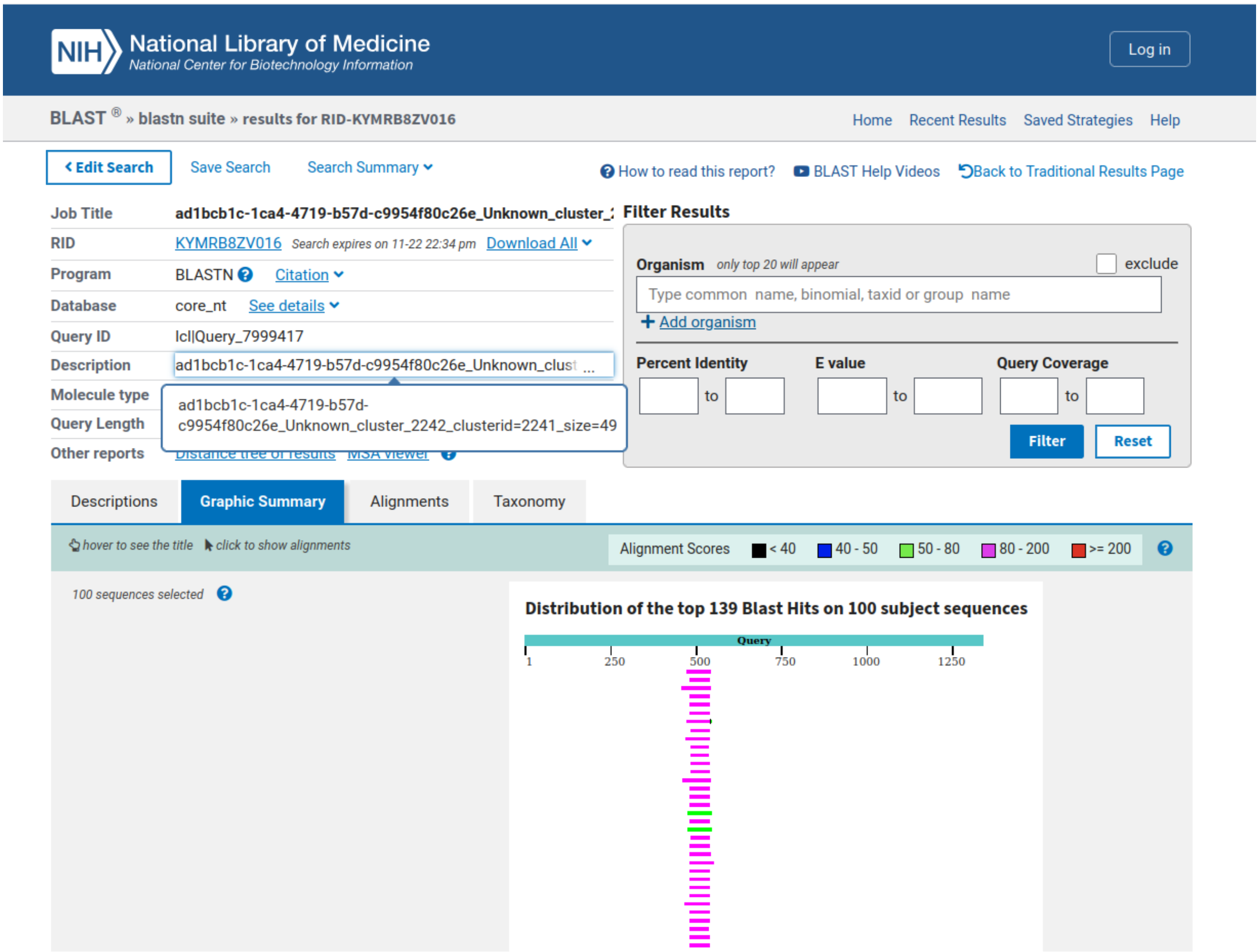

Figure 8: Cluster 2241 Graphical alignments blastn vs nr

These kind of sequences are puzzling and difficult to interpret. Chimera ? Strange organisms ?

Cluster 1108

>ad1bcb1c-1ca4-4719-b57d-c9954f80c26e_Unknown_cluster_1108_clusterid=1107_size=43

GTTTGATCATGGCTCAGGCAATTCTGGCCGAAGGCTTGAAGAACATAGCGGCAGGCGCGAAT
CCGATGCTGTTACGTACGGGTTTGGAGCTTGGGGTAGAGGCATTGGTGGAAGAGATCAAGGC
CATGAGCAAATCCATCGAGACGCACGAAGAGATTGCACAGGTCGCCACGATAGCCTCCGGTG
ATCCCGATATTGGTGAAATGATTGCTGAAGTACTCGATCAGGTGGGTAGAGACGGCATCGTTG
TGGTAGAAGAAGGGCAGGGCTTGGTCTCAGAGATAGAGTATATCAAGGGGCTAAAGTTTGAC
CGTGGCTACATTTCCCCCTCCATGGCAAACGATCAAGAACACATGGAAGCTGTACTATCGAAC
CCCTCTATCCTGATTACCGACAAGAAGATCACCACCATTACAGAACTGCTCCCGCTGCTAGAA
CGTGTGTTAGCAACAGGCAAGAAAGAGGTGCTGGTTATTGCTGAGGATATCGTGGGAGAAGC
CCTGGCTACGCTGGTGGTCAACAAAATGCGTGGAACCTTTTATGTTTTAGGAGTGAGAGCACC
AAGCTTTGGTGACCGTCGTGAAGCGATGATGGAAGATATCGCCATCATGACAGGAGGGCGTG
TCATCAGCGAGAAGGTAGGTCTCAGGCTAGAGAAAGCTACTTTGAAAGACGTGGGGACTGCT
CACAGCGTAACCACGACAAAAGACTCTACGTTGATCGTAGACGGCGGCGGAAGTAAAACGG
CTATTCAGTCGCGCATACGCGAACTCCGCGCCCTGCTTGCTAACGGCGCGAGTGACTATGACC
GCGAGAAGTTGCAAGAGCGGCTGGCAAATTTAACAGGTGGCGTTGGTGTAATCAAAGTGGGG
GCCGCTACCGAGGTTGAGATGAAGGAGAAGAAGCTGCGCGTGCAAGACGCATTGTCGGCAA
CACGCGCTGCTCTCGAAAGGGGTTGTTCCTGGTGGCGGTGCAGCCCTGGTGATTGCACTTCC
GGCCCTGGACAACATCAAGACAAAGATGCCAGAAGAAATGACAGGGGTAAATATCCTGCGGC
GTGCCCTGGAAGAGCCTCTACGTCAGATCGCTGTTAACGCCGGATATGATGGCTCTGTAGTCG
TTGCCAATGTTCGCGAAAAACCGTTTGGATACGGCTTTGATGCGACCTGCGGTCAGTATGTAG
ATATGTTCAAAGCTGGCATCGTCGATCCCGTGAAGGTAACGCGGGCTGCCCTACAAAATGCCG
TGAGTATCGCAGCCCTGCTACTAACAACAGACACACTTATCACTGATAAGCCGGCTCATATTCA
GGTTTCAAAGATATCGAGTTTGGCCTGTAAGGGAGAGTCGCAAGTCGTAACAAGGTAAC

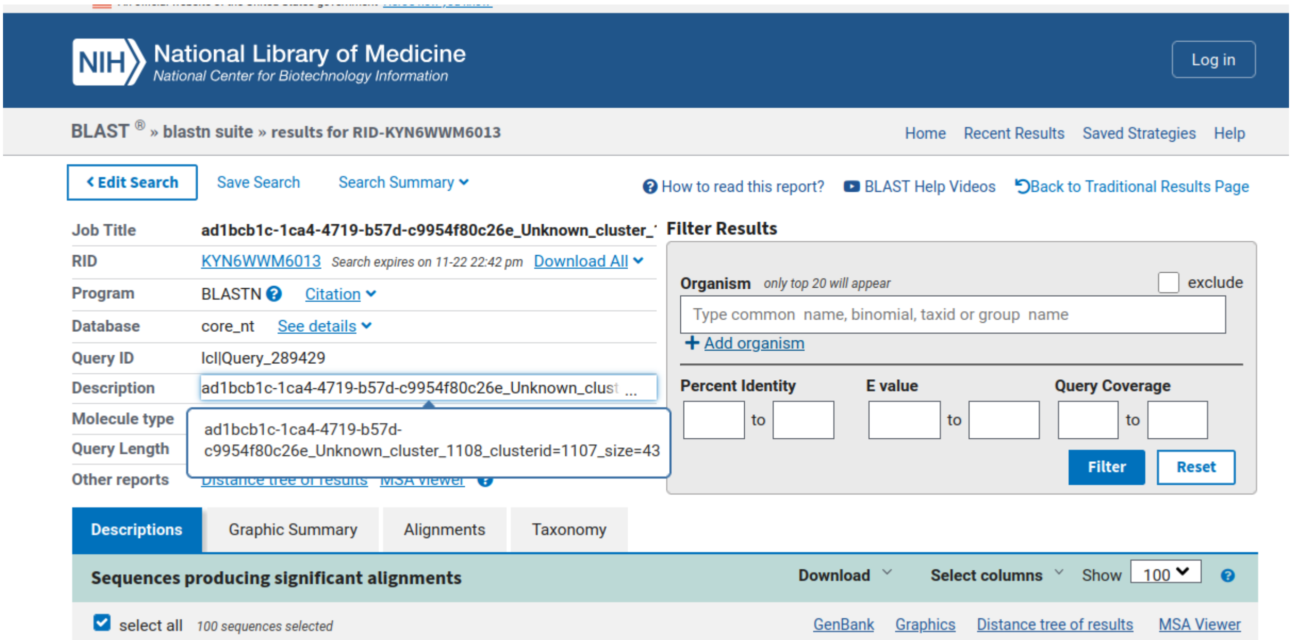

	Description	Scientific Name	Max Score	Total Score	Query Cover	E value	Per. Ident	Acc. Len	Accession
☑	Thermosporothrix sp. COM3 DNA, nearly complete genome	Thermosporothrix sp. ...	418	736	93%	2e-111	67.85%	7674705	AP019376.1
☑	Ktedonobacteria bacterium brp13 DNA, complete genome	Ktedonobacteria bacte...	319	472	93%	1e-81	65.79%	8879929	AP024096.1
☑	Roseiflexus sp. RS-1, complete genome	Roseiflexus sp. RS-1	198	328	70%	3e-45	66.06%	5801598	CP000686.1
☑	Heliorestis convoluta strain HH chromosome, complete genome	Heliorestis convoluta	187	187	49%	5e-42	66.96%	3218981	CP045875.1
☑	Paenibacillus brasilensis strain KACC 13842 chromosome, complete genome	Paenibacillus brasilensis	185	256	60%	2e-41	72.51%	5608532	CP045298.1
☑	Paenibacillus polymyxa strain C12 chromosome, complete genome	Paenibacillus polymyxa	181	181	59%	2e-40	65.20%	5793823	CP023711.1
☑	Paenibacillus polymyxa strain MEZ6 chromosome, complete genome	Paenibacillus polymyxa	181	181	59%	2e-40	65.20%	5769542	CP086373.1
☑	Paenibacillus sp. S02 chromosome, complete genome	Paenibacillus sp. S02	179	179	72%	8e-40	64.93%	6060529	CP073682.1
☑	Paenibacillus polymyxa strain R 5.31 chromosome, complete genome	Paenibacillus polymyxa	179	179	72%	8e-40	64.83%	5867087	CP097767.3
☑	Paenibacillus polymyxa strain P3 chromosome, complete genome	Paenibacillus polymyxa	178	178	23%	3e-39	72.70%	5895306	CP113973.1
☑	Paenibacillus peoriae strain ZF390 chromosome, complete genome	Paenibacillus peoriae	178	178	83%	3e-39	64.41%	6193667	CP061172.1
☑	Paenibacillus peoriae strain JJ21 chromosome, complete genome	Paenibacillus peoriae	178	178	23%	3e-39	72.70%	6163013	CP132974.1

Figure 9: Cluster 1108 blastn vs nr results

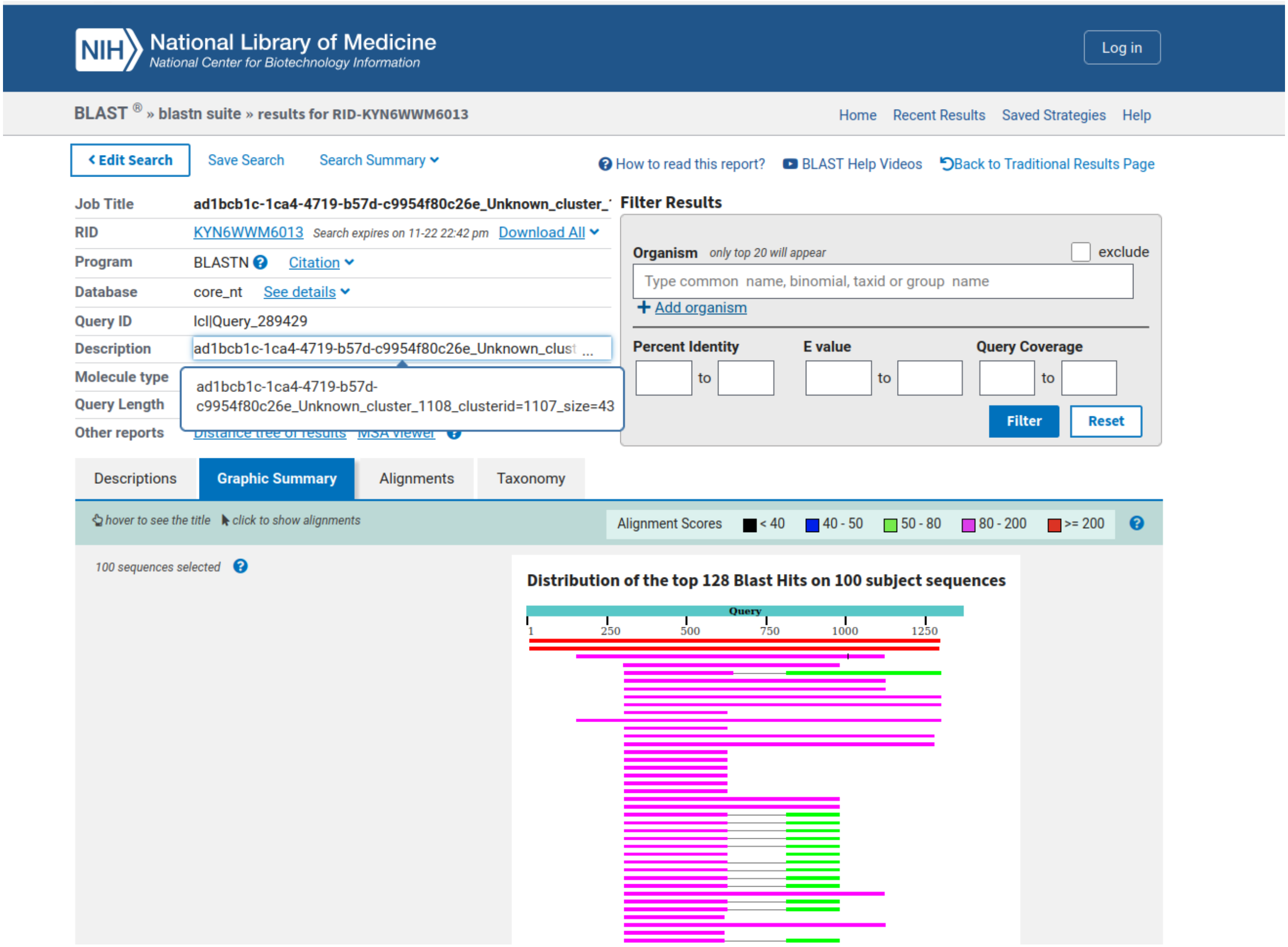

Figure 10: Cluster 1108 graphical alignements blastn vs nr

Our cluster consensus sequence matches with some subjects on 93% of its length.

Those three reference sequences belongs to the Chloroflexota phylum.

ANI with these 3 sequences is around 67%.

If our sequence is not a spurious creation of the wet or dry lab, it could mean that it belongs to an unknown Bacterial Phylum which closest branch is Chloroflexota.

Allegations about phylum discovery should be supported with more data.

But still, this sequence, observed 43 times within this sequencing assay seems to be very far away from what we know while having a complete structure.

Interesting.
